# Enhanced Sonodynamic Therapy and Radiotherapy Efficacy: Modified Polyethylene Glycol–Bismuth Trioxide Nanoplatform for Targeted Tumor Treatment

**DOI:** 10.34133/bmr.0325

**Published:** 2026-02-18

**Authors:** Lanlan You, Mingyuan Dai, Changhao Dong, Min Zheng, Kexin Zhang, Haitao Ran, Jian Liu, Peng Luo, Qin Zhang, Hang Zeng, Jun Wei, Sijing Yan, Yang Yang, Zhigang Wang, E Wen

**Affiliations:** ^1^ Department of Ultrasound, The Second Affiliated Hospital of Chongqing Medical University, Chongqing 400010, China.; ^2^ Department of Ultrasound, The First Affiliated Hospital of Chengdu Medical College, Chengdu 610500, China.; ^3^ Chongqing Key Laboratory of Ultrasound Molecular Imaging, Institute of Ultrasound Imaging, The Second Affiliated Hospital of Chongqing Medical University, Chongqing 400010, China.; ^4^ Shanghai University of Engineering Science, Shanghai 201620, China.; ^5^ Department of Radiology, Chongqing Traditional Chinese Medicine Hospital, Chongqing 400021, China.; ^6^ Department of Ultrasound, Chongqing Health Center for Women and Children/Women and Children’s Hospital of Chongqing Medical University, Chongqing 401147, China.; ^7^ Department of Ultrasound, Chongqing Traditional Chinese Medicine Hospital, Chongqing 400021, China.; ^8^ Precision Medicine Center, The Second Affiliated Hospital of Chongqing Medical University, Chongqing 400010, China.

## Abstract

This study develops a novel multifunctional nanoplatform, modified polyethylene glycol–bismuth trioxide (mPEG-Bi_2_O_3_), synthesized via vacuum ball milling followed by ultrasonic liquid-phase exfoliation and surface PEGylation, to enhance the synergistic effects of sonodynamic therapy (SDT) and radiotherapy (RT). Characterization revealed that mPEG-Bi_2_O_3_ exhibits a thin-layered nanosheet structure (hydrodynamic size: 239.28 ± 4.32 nm; lattice spacing: 0.29 nm) and a zeta potential of −33.64 ± 0.80 mV. Notably, the nanoplatform demonstrated exceptional colloidal stability in physiologically relevant media, maintaining consistent size and surface charge over 7 d in serum-containing medium, which confirms the effectiveness of the PEG coating for biomedical applications. XPS analysis confirmed a mixed Bi^3+^/Bi^5+^ oxidation state, and deconvolution of the O 1s spectrum quantified the oxygen vacancy content at 11.02%, confirming a defect-rich structure. Successful PEG grafting was verified by Fourier transform infrared spectroscopy and quantified by thermogravimetric analysis, showing a grafting content of ~13.59 wt %. Under low-intensity focused ultrasound (LIFU), mPEG-Bi_2_O_3_ significantly enhanced reactive oxygen species generation, leading to a marked reduction in intracellular glutathione levels. In vitro cytotoxicity studies demonstrated favorable selectivity, with lower toxicity toward normal endothelial cells compared to 4T1 cancer cells, and the combination of mPEG-Bi_2_O_3_ and LIFU induced apoptosis in 4T1 cells. In vivo studies showed that intravenous administration of mPEG-Bi_2_O_3_ in tumor-bearing mice resulted in peak tumor accumulation at 24 h (0.17 ± 0.03 %ID/g), correlating with a significant 87.82% ± 4.77% reduction in tumor volume after 14 d of treatment when combined with LIFU and RT (10 Gy), superior to dual-modality treatments. Immune profiling indicated enhanced dendritic cell maturation, increased tumor-infiltrating CD8^+^ T cells, and reduced regulatory T cells, demonstrating immune microenvironment remodeling. Collectively, mPEG-Bi_2_O_3_ presents a surface-engineered strategy for potent SDT-RT synergy with demonstrated biosafety, showing promising potential for solid tumor treatment.

## Introduction

Sonodynamic therapy (SDT) activates sonosensitizers via ultrasound to generate reactive oxygen species (ROS), inducing oxidative stress to kill tumor cells [1–4]. RT, on the other hand, utilizes x-rays to ionize or oxidize tissues, leading to excessive ROS that directly damage tumor DNA and inhibit cellular proliferation [5–8]. The combination of SDT and RT can therefore facilitate additive ROS generation, potentially enhancing tumor-killing efficacy while reducing RT dose and frequency [9–13]. However, achieving effective synergy remains a challenge, often limited by inefficient tumor accumulation, rapid clearance, and insufficient ROS generation of therapeutic agents.

Nanotechnology offers a powerful strategy to overcome these barriers by enabling the design of multifunctional platforms capable of targeted delivery and combined therapies. In this context, inorganic nanomaterials with high atomic numbers are particularly promising. Bismuth (Bi), for instance, exhibits strong x-ray attenuation, which can locally enhance radiation energy deposition at tumor sites, thereby improving RT efficacy while sparing normal tissues [14,15]. Furthermore, bismuth trioxide (Bi_2_O_3_) has emerged as an efficient inorganic sonosensitizer capable of generating ROS under low-intensity focused ultrasound (LIFU) [16,17]. Its sonocatalytic activity can be enhanced by engineering oxygen vacancies to inhibit electron-hole recombination [18,19], thereby maximizing SDT outcomes. Consequently, a prevailing research trend has been to augment the intrinsic properties of Bi-based nanomaterials through sophisticated material engineering, such as constructing heterojunctions or implementing elemental doping [1,9]. However, an equally critical yet underexplored avenue for maximizing in vivo therapeutic efficacy lies in surface engineering to decisively overcome pharmacokinetic and delivery barriers. In this work, we propose that precise surface modification—rather than compositional complexity—can unlock the full therapeutic potential of single-component Bi_2_O_3_. By functionalizing Bi_2_O_3_ with amphiphilic polyethylene glycol (mPEG), we aim to engineer a nanoplatform (mPEG-Bi_2_O_3_) with enhanced colloidal stability, prolonged circulation, and efficient tumor accumulation, all while preserving its inherent radiosensitizing and sonocatalytic activities. We demonstrate that this surface-centric approach not only facilitated enhanced ROS generation under combined LIFU and x-ray irradiation but also promoted effective tumor accumulation and retention. Furthermore, the platform was designed to remodel the tumor immune microenvironment, potentially activating antitumor immunity. Our study thus highlights targeted surface engineering as a powerful and complementary strategy to intrinsic material design, offering a streamlined and potentially more translatable paradigm for achieving SDT-RT synergy. This work provides a compelling perspective for developing efficient and clinically feasible nanoplatforms for combined SDT-RT.

## Materials and Methods

### Preparation of the mPEG-Bi_2_O_3_ nanoplatform

mPEG-Bi_2_O_3_ nanoplatforms were synthesized using vacuum ball milling and ultrasonic liquid-phase exfoliation methods [20,21]. Bi sodium nitrate (1.0 g, Ruiqi Biotechnology, Xi’an, China) was placed in a 100-ml zirconia milling jar with zirconia grinding balls at a ball-to-powder weight ratio of 20:1. The jar was sealed, evacuated to a pressure of −0.1 MPa, and milled at 120 rpm for 8 h. This pretreatment step was performed in 3 independent batches, and the resulting ball-milled powder exhibited high consistency in subsequent characterizations, confirming good reproducibility. Subsequently, 30 mg of the vacuum-ball-milled powder was mixed with 200 mg of mPEG (Ruiqi Biotechnology) in 40 ml of deionized water. The mixture was first dispersed using an ultrasonic cleaning bath for 30 min and then subjected to intense exfoliation and modification using a probe ultrasonic cell disruptor for 6 h. The final product was collected by centrifugation, washed thoroughly with deionized water, and isolated via freeze-drying. The concentration of the obtained mPEG-Bi_2_O_3_ nanosheets was determined by weighing the freeze-dried product, which was then redispersed in deionized water and stored at 4 °C for further use.

### Characterization of the mPEG-Bi_2_O_3_ nanoplatform

The morphology, size, and elemental composition of the mPEG-Bi_2_O_3_ nanoplatform were examined using a transmission electron microscope (TEM) (JEOL JEM-F200, Japan) and a scanning electron microscope (SEM) (ZEISS Sigma 300, Germany). The layer thickness of mPEG-Bi_2_O_3_ was assessed with an atomic force microscope (AFM) (Bruker Dimension FastScan; Bruker Dimension Icon, Germany). The crystalline structure was analyzed via x-ray diffraction (XRD) (SHIMADZU XRD-6100, Japan). To assess colloidal stability under biologically relevant conditions, nanoparticle size and zeta potential were monitored over 7 d. Measurements were conducted in deionized water, phosphate-buffered saline (PBS; simulating physiological ionic strength), and complete RPMI 1640 medium supplemented with 10% fetal bovine serum (FBS) (simulating a protein-rich environment) using a dynamic light scattering (DLS) instrument (Malvern Zetasizer Nano ZS90, UK). X-ray photoelectron spectroscopy (XPS) (Nexsa, ThermoFisher, USA) was utilized to obtain XPS spectra and confirm the presence of oxygen vacancies. Fourier transform infrared spectroscopy (FTIR) (Thermo Scientific Nicolet iS50, USA), with a wavenumber range of 500 to 4,000 cm^−1^, was employed to gather structural information on mPEG-Bi_2_O_3_. XPS data analysis and peak deconvolution were performed using XPSPEAK 4.1 software. Thermogravimetric analysis (TGA) was conducted on the Mettler–Toledo TGA/DSC1 (Mettler–Toledo, GmbH, Switzerland) under a nitrogen atmosphere with a heating rate of 10 °C /min.

### LIFU parameters and setup

All ultrasound exposures were performed using a custom LIFU system (model D2006, Chongqing Ronghai Ultrasound Medical Engineering Research Center Co. Ltd., China) equipped with a focused transducer (aperture diameter: 10 mm). The acoustic output power was calibrated using a radiation force balance before the experimental series and verified periodically.

In vitro experiments were conducted with the following parameters: a fundamental frequency of 1.0 MHz, a spatial average intensity of 1.5 W/cm^2^, a duty cycle of 50% in pulsed mode with a pulse repetition frequency of 1 kHz, and an exposure duration of 2 min per well. For cellular treatments, the transducer was positioned beneath the culture plate, coupled through a sterile ultrasound-conducting hydrogel pad (thickness: 1.5 mm). This setup placed the cell monolayer at an approximate distance of 1.5 mm from the transducer surface, with degassed water as the coupling medium within the system.

In vivo tumor treatment was performed using the same transducer at a frequency of 1.0 MHz, an intensity of 2.5 W/cm^2^, and a duty cycle of 50% (pulse repetition frequency: 1 kHz) in pulsed mode, for a duration of 3 min per tumor per session. Mice were anesthetized, and the tumor area was shaved. An ample ultrasound transmission gel was applied to ensure acoustic coupling. The transducer was then placed in gentle, direct contact with the skin surface over the center of the tumor and held stationary during sonication. To prevent potential thermal accumulation, the transducer temperature was monitored. If any temperature increase was detected, an ice–water mixture was applied intermittently to the skin–transducer interface for cooling, ensuring that the treatment remained nonthermal.

### In vitro verification of ROS generation, glutathione consumption, and oxygen production of mPEG-Bi_2_O_3_

For measurement of ROS generation, different concentrations of mPEG-Bi_2_O_3_ (0, 300, 600, 900, 1,200, 1,500, and 1,800 μg/ml) at 60 μl were incubated with 3,3′,5,5′-tetramethylbenzidine (TMB) (8.3 mM, 20 μl) and hydrogen peroxide (H_2_O_2_) at pH 5.5 (31.25 μM, 400 μl). After incubation for 10 min, 100 μl of the reaction solution from each concentration group was transferred into a 96-well plate, and the absorbance was measured at wavelengths from 300 to 800 nm using a SpectraMax ID3 microplate reader (Molecular Devices, San Jose, CA, USA). The 2′,7′-dichlorodihydrofluorescein diacetate (DCFH-DA) (Beyotime, Shanghai, China) was used as a fluorescent probe to detect ROS generation.

For discoloration analysis, 20 parts per million (ppm) Bi_2_O_3_ and 5 ppm 1,3-diphenylisobenzofuran (DPBF) were dissolved in dimethyl sulfoxide at pH 7.3. LIFU was performed as described above. The reaction was conducted at 37 °C for 30 min.

The ultraviolet-visible spectroscopy was conducted to determine glutathione (GSH) consumption. Briefly, 1,600 μg/ml of mPEG-Bi_2_O_3_ nanoparticles was mixed with 800 μM GSH and 10 mM dithio-bis-nitrobenzoic acid (DTNB) in PBS solution (pH 7.4). At various time points (0, 2, 4, 8, 20, 24, and 48 h), the absorbance at 412 nm was measured on a SpectraMax iD3 Multi-Mode Microplate Reader (Molecular Devices, USA).

Additionally, the oxygen production of mPEG-Bi_2_O_3_ was also measured. In detail, to simulate the slightly acidic tumor environment and normal physiological pH conditions, 2 groups were set at pH values of 6.0 and 7.4, with amounts of PBS and H_2_O_2_ as follows: 3 ml and 50 μl (1.747 mM), respectively. Each group was further divided based on the presence or absence of mPEG-Bi_2_O_3_, with a concentration of 1,600 μg/ml (60 μl). After thoroughly mixing the reagents, the oxygen content was measured using a JPB-607A portable dissolved oxygen meter (Shanghai INESA Scientific Instrument Co. Ltd., China), recording data every 10 s.

### ESR detection of ROS types

The generation of ROS was detected by electron spin resonance (ESR) spectroscopy using specific spin traps. The superoxide anion (·O_2_^−^) was captured using TEMPO (2,2,6,6-tetramethylpiperidine-1-oxyl), and the hydroxyl radical (·OH) was captured using DMPO (5,5-dimethyl-1-pyrroline-N-oxide). For the assay, mPEG-Bi_2_O_3_ at a final concentration of 100 μg/ml was mixed with the respective spin trap in the presence or absence of H_2_O_2_ (100 μM). The mixture was then subjected to LIFU irradiation. Five experimental groups were set up: mPEG-Bi_2_O_3_ + LIFU + H_2_O_2_, mPEG-Bi_2_O_3_ + LIFU, mPEG-Bi_2_O_3_ + H_2_O_2_, H_2_O_2_ alone, and mPEG-Bi_2_O_3_ alone. After treatment, the ESR signals were immediately detected using an ESR spectrometer (Bruker Magnettech ESR5000, Germany).

### Cell culture

The 4T1 breast cancer cells and human umbilical vein endothelial cells (HUVECs) were obtained from the Institute of Ultrasound Imaging at Chongqing Medical University. The 4T1 breast cancer cells were cultured in fresh 4T1 medium (Procell, Wuhan, China) containing 10% FBS and 1% penicillin/streptomycin, while HUVECs were cultured in Dulbecco’s modified Eagle’s medium (Gibco, USA) supplemented with 10% FBS, 1% penicillin/streptomycin, and 30 μg/ml endothelial cell growth supplement (ScienCell, USA). All cells were maintained at 37 °C in a 5% CO_2_ incubator.

### Cell Counting Kit-8 assay

Cell viability was assessed using the Cell Counting Kit-8 (CCK-8) method to evaluate the cytotoxicity of mPEG-Bi_2_O_3_ at different concentrations and to select the optimal therapeutic concentration. In detail, 4T1 cells and HUVEC cells (both 100 μl, 5 × 10^4^ cells/ml) were seeded into each well of a 96-well plate and incubated for 24 h. Then, the cells were incubated with different concentrations of mPEG-Bi_2_O_3_ (0, 6.25, 12.5, 50, 100, and 200 μg/ml) for an additional 24 h. Finally, the absorbance at 450 nm was measured after incubation with CCK-8 (Beyotime, China). The cell viability was calculated.

### mPEG-Bi_2_O_3_ uptake

The uptake of mPEG-Bi_2_O_3_ by cells was monitored using a confocal laser scanning microscope (CLSM). Briefly, mPEG-Bi_2_O_3_ was first labeled with 1,1′-dioctadecyl-3,3,3′,3′-tetramethylindocarbocyanineperchlorate (Dil) at room temperature for 12 h. Then, 4T1 cells in the logarithmic growth phase were cultured overnight in confocal dishes, and then the medium was replaced with medium containing Dil-labeled mPEG-Bi_2_O_3_ (50 μg/ml). After different time intervals (0, 0.5, 1, 2, and 4 h), cells were washed with PBS and immediately fixed with 4% paraformaldehyde for 15 min. The cell nuclei were stained with 4′,6-diamidino-2-phenylindole (DAPI). Finally, the cells were observed under the CLSM (Andor Dragonfly 200, Andor Technology, UK) and analyzed on a flow cytometer (CytoFLEX, Beckman Coulter, USA).

### In vitro therapeutic effects of SDT

To evaluate the in vitro SDT effects of mPEG-Bi_2_O_3_, flow cytometry detection of apoptosis was first performed. Briefly, 4T1 cells (5 × 10^5^ cells/ml) were seeded in 6-well plates and incubated at 37 °C for 24 h. The cells were then divided into 4 groups: control (untreated) group, LIFU group, mPEG-Bi_2_O_3_ group, and mPEG-Bi_2_O_3_ + LIFU group. Cells in the mPEG-Bi_2_O_3_ and mPEG-Bi_2_O_3_ + LIFU groups were incubated with mPEG-Bi_2_O_3_ (200 μg/ml) for 12 h. Subsequently, cells in the LIFU and mPEG-Bi_2_O_3_ + LIFU groups were irradiated with LIFU. After treatment, cells were collected, and Annexin V–fluorescein isothiocyanate (FITC)/DAPI staining was performed according to the kit instructions (Beyotime). Finally, cell apoptosis was assessed using a CytoFLEX flow cytometer (Beckman Coulter). Additionally, the above treatment with mPEG-Bi_2_O_3_ + LIFU was repeated, and after calcein-AM/propidium iodide (PI) staining (1:1,000 dilution; Beyotime), dead cells (red fluorescence) and live cells (green fluorescence) were distinguished using CLSM. GSH consumption in each group was calculated according to the instructions of the GSH (Solarbio, Beijing, China) and oxidized glutathione disulfide (GSSG) detection kit (Beyotime), with the blank group’s GSH content set at 100%. JC-1 staining was used to detect changes in mitochondrial membrane potential, and intracellular ROS was detected using the fluorescent probe DCFH-DA.

### Study animals

Female specific pathogen-free BALB/c mice (7 to 8 weeks old) were obtained from Chongqing Enbi Biotechnology Co. Ltd. (Chongqing, China) and were housed in a controlled environment (22 to 27 °C, 50% to 70% humidity, 12-h/12-h light/dark cycle). All animal experiments were approved by the Ethics Committee of The Second Affiliated Hospital of Chongqing Medical University (approval no. IACUC-SAHCQMU-2024-00092).

### Establishment of the 4T1 tumor-bearing mouse model

To establish subcutaneous tumor models in BALB/c female mice, 100 μl of a 4T1 cell suspension (1 × 10^6^ cells per mouse) was injected subcutaneously. Seven days later, tumors that reached a volume of 100 mm^3^ were selected for subsequent experiments.

### mPEG-Bi_2_O_3_-mediated antitumor effects in vivo

BALB/c mice with established subcutaneous 4T1 tumors were randomly assigned to 7 groups (*n* = 5 per group): control, LIFU alone, mPEG-Bi_2_O_3_, mPEG-Bi_2_O_3_ + LIFU, RT alone, mPEG-Bi_2_O_3_ + RT, and mPEG-Bi_2_O_3_ + LIFU + RT. mPEG-Bi_2_O_3_ was administered via the tail vein at a concentration of 2 mg/ml and a volume of 200 μl (20 mg/kg), followed by LIFU treatment 4 h post-injection. After LIFU, RT was administered using an x-ray irradiator (Elekta Infinity, Elekta AB, Stockholm, Sweden) at a single dose of 10 Gy. The control group was administered saline without LIFU or x-ray irradiation. Tumor volume and body weight were recorded every 2 d until the tumor volume exceeded 1,000 mm^3^. The study endpoint for survival analysis was defined as either (a) the natural death of the animal or (b) the point at which the tumor volume exceeded 1,500 mm^3^ or showed signs of ulceration, at which time the mouse was humanely euthanized and recorded as deceased for survival analysis. Survival data were recorded as the number of days from the initiation of treatment until the defined endpoint. Tumor volume was calculated using the following formula: Volume = *W*^2^ × *L*/2 (where *W* is the width and *L* is the length). The tumor inhibition rate (%) was calculated as [1 − (tumor volume of treatment group / tumor volume of control group)] × 100%. For morphological structure and apoptosis/proliferation analysis, one BALB/c nude mouse was randomly selected from each group 24 h after treatment and euthanized by cervical dislocation, and tumor tissue was collected for ROS, hematoxylin and eosin (H&E) staining, terminal deoxynucleotidyl transferase-mediated deoxyuridine triphosphate nick end labeling (TUNEL), and Ki-67 staining.

### Evaluation of antitumor immune effects in vivo

On the eighth day post-treatment, lymph nodes and tumor tissues were collected for the preparation of single-cell suspensions. After labeling the lymph node single-cell suspensions with anti-CD11c FITC, anti-CD86 PE (phycoerythrin), and anti-CD80 APC (allophycocyanin) antibodies, flow cytometry was conducted to investigate dendritic cell (DC) maturation. To further evaluate the distribution ratio of cytotoxic T lymphocytes (CD8^+^) and regulatory T cells (Tregs) in the tumor microenvironment, the staining with anti-CD3 PE/anti-CD4 FITC/anti-CD8 APC or anti-CD3 PE/anti-CD4 FITC/anti-Forkhead box P3 (FoxP3) PC5.5 was performed. All antibodies used in the experiments were purchased from BioLegend.

Serum samples were collected, and cytokines, including interleukin-6 (IL-6), tumor necrosis factor-α (TNF-α), IL-10, IL-12p70, and interferon-γ (IFN-γ), were analyzed using the corresponding enzyme-linked immunosorbent assay (ELISA) kits (Jiangsu Sumeike Biological Technology Co. Ltd., China).

### In vivo biodistribution of mPEG-Bi_2_O_3_

After tail vein injection of mPEG-Bi_2_O_3_ into 4T1 tumor-bearing mice, the animals were euthanized at various time points (0, 1, 2, 6, 12, and 24 h post-injection). The tumors, heart, liver, spleen, lungs, and kidneys were excised, rinsed with saline, weighed, and recorded. Tissues were then digested in aqua regia (a 3:1 mixture of hydrochloric acid and nitric acid) for 2 d, followed by heating at 75 °C for 1 d. After centrifugation and dilution with 2% diluted nitric acid, the metabolic pathways and distribution of mPEG-Bi_2_O_3_ in tumors and organs were analyzed using inductively coupled plasma optical emission spectroscopy/mass spectrometry (ICP-OES/MS) (Agilent 7700, USA). A standard curve was prepared using a certified Bi standard solution for absolute quantification. The Bi content in each tissue was first calculated as micrograms of Bi per gram of wet tissue weight (μg/g). For pharmacokinetic analysis, this value was converted to the percentage of injected dose per gram of tissue (%ID/g) using the following formula: %ID/g = (Bi content in tissue (μg)/Injected dose of Bi (μg)) × 100%/Tissue weight (g). The injected dose of Bi for each animal was precisely calculated based on the concentration of the administered mPEG-Bi_2_O_3_ suspension and the individual animal’s body weight.

### Evaluation of the biosafety and biocompatibility of mPEG-Bi_2_O_3_

Healthy BALB/c mice (*n* = 27) were tail vein injected with different doses of mPEG-Bi_2_O_3_ (10, 20, and 40 mg/kg). Blood samples were collected on days 1, 14, and 28 for hematological analysis and biochemical testing. The control group of mice was given 200 μl of PBS using the same administration method. The heart, liver, spleen, lung, and kidney were fixed in 4% paraformaldehyde and subsequently subjected to pathological analysis, including H&E staining.

### Statistical analysis

All data were expressed as means ± standard deviation (SD). For comparisons between 2 groups only, an unpaired, 2-tailed Student’s *t* test was applied. For comparisons across 3 or more groups, one-way analysis of variance (ANOVA) followed by Tukey’s post hoc test for multiple comparisons was applied. Survival data were analyzed using the Kaplan–Meier method, and comparisons between groups were performed with the log-rank test. A *P* value of <0.05 was considered statistically significant. Statistically significant differences are denoted as *P ≤ 0.05, **P ≤ 0.01, and ***P ≤ 0.001.

## Results

### Characterization of mPEG-Bi_2_O_3_

**Fig. 1.**
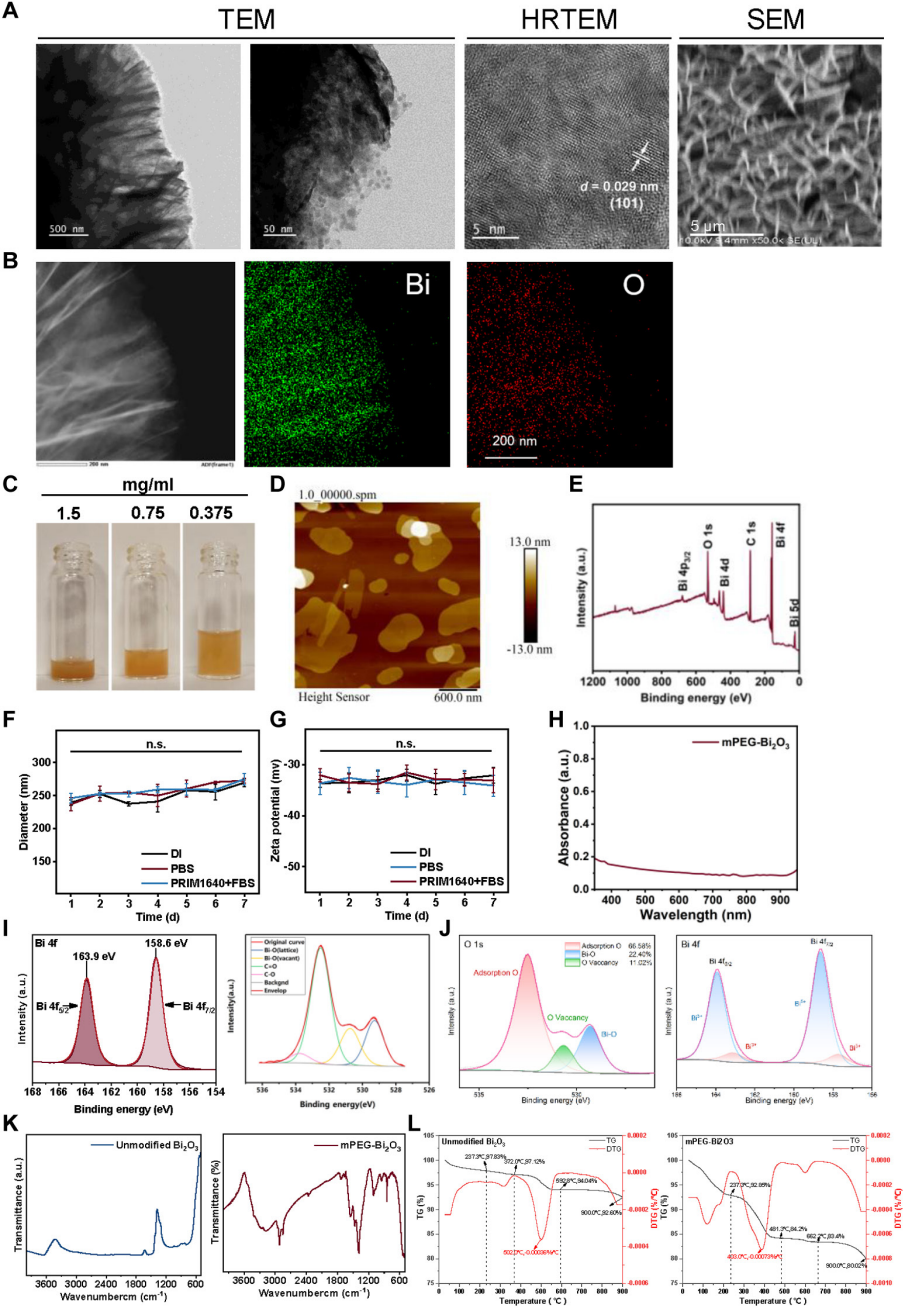
Characterization of mPEG-Bi_2_O_3_. (A) TEM, high-resolution TEM (HRTEM), and SEM images of mPEG-Bi_2_O_3_ (scale bars, 500 nm, 20 nm, 5 nm, and 5 μm). (B) Energy spectrum of Bi and O in mPEG-Bi_2_O_3_ (scale bar, 200 nm). (C) Digital photograph of the mPEG-Bi_2_O_3_ suspension. (D) AFM images of mPEG-Bi_2_O_3_. (E) XRD pattern of mPEG-Bi_2_O_3_. (F) Hydrodynamic diameter of mPEG-Bi_2_O_3_ monitored over 7 d in deionized (DI) water, phosphate-buffered saline (PBS), and RPMI 1640 medium supplemented with 10% fetal bovine serum (RPMI 1640 + FBS). (G) Zeta potential of mPEG-Bi_2_O_3_ monitored over 7 d in DI, PBS, and RPMI 1640 + FBS. (H) Ultraviolet–visible–near-infrared absorption spectrum of mPEG-Bi_2_O_3_. (I) High-resolution XPS spectrum of Bi 4f. (J) Deconvoluted high-resolution XPS spectrum of O 1s, showing lattice oxygen (Bi–O), oxygen vacancy, and surface oxygen components. (K) Comparative FTIR spectra of unmodified Bi_2_O_3_ and mPEG-Bi_2_O_3_. (L) Thermogravimetric (TG) and derivative thermogravimetric (DTG) curves of unmodified Bi_2_O_3_ and mPEG-Bi_2_O_3_. Data in (F) and (G) are presented as mean ± SD (*n* = 3 independent measurements). Statistical analysis by one-way ANOVA showed no significant differences (n.s.) in size or zeta potential across media or over the 7-d period.

TEM and SEM analyses revealed that the synthesized mPEG-Bi_2_O_3_ displayed a thin-layered nanosheet structure with apparent porosity, and the interlayer spacing was 0.29 nm (Fig. 1A). They showed good dispersion (Fig. 1B) and appeared as a brownish yellow suspension (Fig. 1C). AFM analysis indicated that mPEG-Bi_2_O_3_ consisted of thin-layer nanosheets (Fig. 1D). The XRD pattern demonstrated that the prepared mPEG-Bi_2_O_3_ was composed of oxygen (O) and Bi (Fig. 1E). DLS measurements revealed that the average hydrodynamic diameter of mPEG-Bi_2_O_3_ in deionized water was approximately 239.28 ± 4.32 nm (Fig. 1F), and the zeta potential was about −33.64 ± 0.80 mV (Fig. 1G). Critically, the colloidal stability of the nanoplatform was systematically evaluated under physiologically relevant conditions. Over a 7-d period, mPEG-Bi_2_O_3_ maintained consistent hydrodynamic sizes and high negative surface charges in PBS and RPMI 1640 medium supplemented with 10% FBS. Specifically, in the serum-containing medium, the hydrodynamic diameter remained stable (246.18 ± 6.91 nm on day 1 versus 275.10 ± 8.33 nm on day 7), and the zeta potential showed only minimal variation (−32.05 ± 1.28 mV on day 1 versus −33.07 ± 2.41 mV on day 7), with no statistically significant differences observed across the 3 media or over time. This demonstrates exceptional resistance to aggregation induced by high ionic strength and the presence of serum proteins, confirming the effectiveness of the mPEG coating in providing steric stabilization. The ultraviolet-visible spectroscopy suggested that Bi_2_O_3_ exhibited full-spectrum absorption within the wavelength range of 200 to 1,100 nm, with no characteristic absorption peaks (Fig. 1H). To elucidate the surface chemistry and composition, XPS and thermal analyses were performed. XPS analysis of the Bi 4f region indicated the coexistence of Bi^3+^ and Bi^5+^ oxidation states (Fig. 1I). Further deconvolution of the high-resolution O 1s spectrum (Fig. 1J) revealed 3 components corresponding to lattice oxygen (Bi–O, 529.31 eV), oxygen vacancies (530.67 eV), and surface-adsorbed oxygen (532.51 eV). The relative content of oxygen vacancies was quantified to be 11.02%, confirming the defect-rich nature of the material. FTIR spectroscopy was used to verify the surface modification. Comparative analysis between unmodified Bi_2_O_3_ and mPEG-Bi_2_O_3_ (Fig. 1K) clearly shows the emergence of characteristic peaks in the latter, most notably the strong C–O–C stretching vibration at ~1,100 cm^−1^, which was absent in the spectrum of unmodified Bi_2_O_3_. TGA (Fig. 1L) was used to estimate the loading of surface-grafted mPEG. The distinct weight loss steps between 237 °C and 481 °C in the mPEG-Bi_2_O_3_ curve, attributed to mPEG decomposition, corresponded to a grafting content of approximately 13.59 wt %. Together, the distinctive FTIR signature, quantitative TGA data, high negative zeta potential, and exceptional stability in complex media provide conclusive and multifaceted evidence for the successful grafting of mPEG onto the Bi_2_O_3_ surface.

### ROS generation and catalytic efficiency of mPEG-Bi_2_O_3_ under LIFU irradiation

**Fig. 2.**
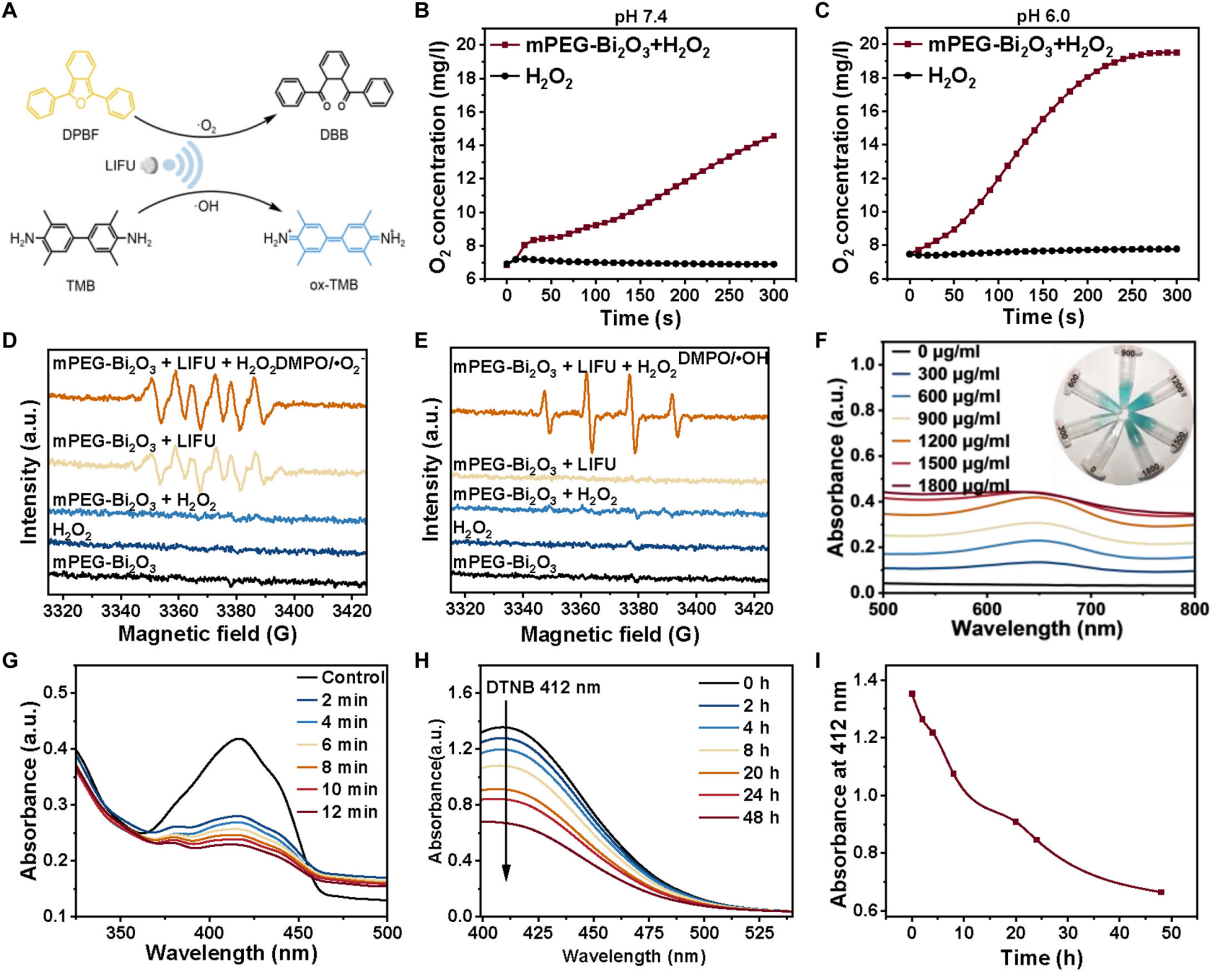
Sonocatalytic performance of mPEG-Bi_2_O_3_. (A) Schematic diagram of ROS generation by mPEG-Bi_2_O_3_ under LIFU. (B) Catalytic oxygen generation by mPEG-Bi_2_O_3_ in a simulated physiological environment (pH 7.4). (C) Catalytic oxygen generation in a simulated tumor acidic environment (pH 6.0). (D) ESR spectra detecting •O_2_^−^ using TEMPO. (E) ESR spectra detecting •OH using DMPO. (F) Time-dependent oxidation of TMB indicating ROS generation. (G) Degradation of DPBF under LIFU irradiation. (H) Time-dependent depletion of GSH monitored by the DTNB assay. (I) Decay of the characteristic DTNB absorbance at 412 nm. Data are representative of 3 independent experiments.

Under LIFU irradiation, mPEG-Bi_2_O_3_ demonstrated excellent sonocatalytic performance, effectively generating ROS (Fig. 2A). To quantitatively validate the proposed cascade mechanism of “oxygen enhancement–ROS burst–antioxidant barrier disruption”, we conducted a series of kinetic measurements. In a simulated tumor microenvironment (pH 6.0, with H_2_O_2_), dissolved oxygen monitoring showed that mPEG-Bi_2_O_3_ rapidly catalyzed H_2_O_2_ decomposition to produce oxygen, with a significant increase in solution oxygen content within 10 min (Fig. 2B and C). This result confirms its efficient “oxygen enhancement” capability. ESR spectroscopy distinctly captured characteristic signals of ·O_2_^−^ and ·OH, with the highest signal intensity observed in the mPEG-Bi_2_O_3_ + LIFU group (Fig. 2D and E), providing direct spectroscopic evidence for the “ROS burst”. This finding was further corroborated by TMB colorimetric analysis (Fig. 2F) and DPBF degradation experiments, where the characteristic absorption peak decreased by 90% within 12 min under mPEG-Bi_2_O_3_ + LIFU treatment (Fig. 2G). Concurrently, dynamic monitoring via the DTNB assay revealed that mPEG-Bi_2_O_3_ effectively depleted GSH, as indicated by a significant time-dependent decrease in the characteristic absorbance (Fig. 2H and I). This clearly demonstrates the process of “antioxidant barrier disruption”. These systematic quantitative results together constitute the experimental foundation for the proposed synergistic mechanistic model, strongly supporting the significant potential of mPEG-Bi_2_O_3_ in SDT.

### Cytotoxicity, cellular uptake, ROS generation, and in vitro therapeutic effect of mPEG-Bi_2_O_3_

**Fig. 3.**
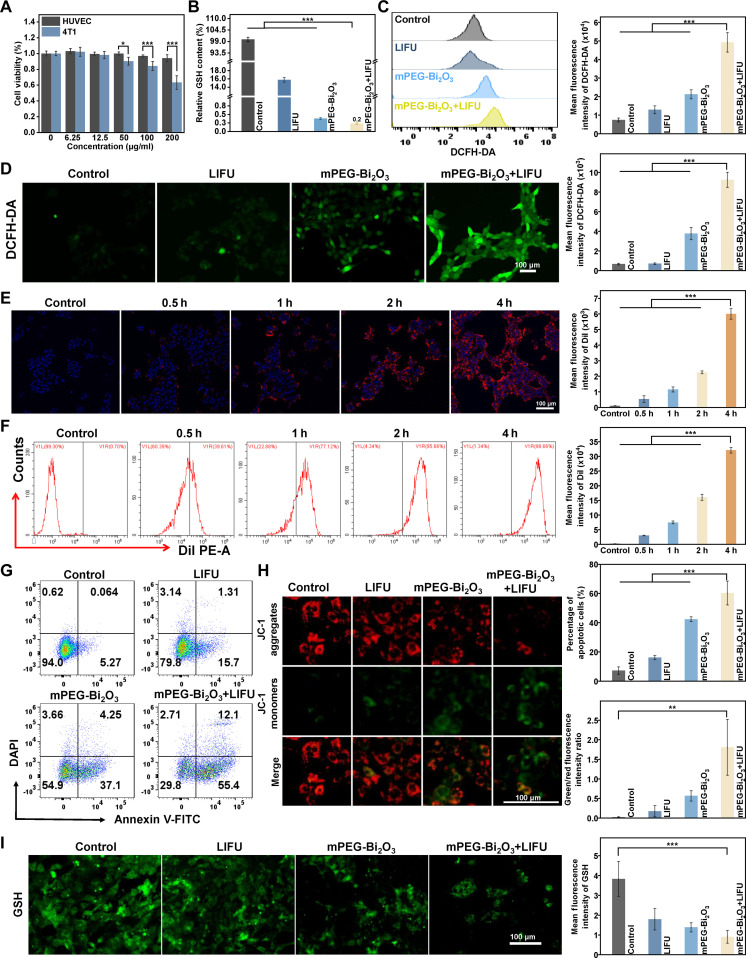
In vitro cytotoxicity, cellular uptake, and sonodynamic therapeutic effects of mPEG-Bi_2_O_3_. (A) Viability of 4T1 breast cancer cells and HUVECs after incubation with different concentrations of mPEG-Bi_2_O_3_, as determined by CCK-8 assay. (B) Quantitative analysis of intracellular GSH depletion under different treatment conditions. (C) Representative flow cytometry histograms (left) and quantitative analysis of mean fluorescence intensity (right). (D) DCFH-DA staining of intracellular ROS generation in 4T1 cells (left) and quantitative analysis of mean fluorescence intensity (right). (E) CLSM images showing the time-dependent cellular association/internalization of Dil-labeled mPEG-Bi_2_O_3_ (red) in 4T1 cells. Nuclei are stained with DAPI (blue). Scale bar, 100 μm. Right panel: Quantitative analysis of mean fluorescence intensity. (F) Flow cytometry analysis of cellular uptake (left) and mean fluorescence intensity of Dil over time (right). (G) Flow cytometry analysis of apoptosis using Annexin V-FITC/PI staining. (H) Representative JC-1 staining images (left) and quantitative analysis of the green/red fluorescence intensity ratio (right), indicating mitochondrial membrane potential depolarization. (I) Quantitative analysis of GSH consumption after different treatments. Data are mean ± SD (*n* = 3 independent experiments). *P < 0.05, **P < 0.01, ***P < 0.001.

To evaluate the therapeutic selectivity of the mPEG-Bi_2_O_3_ nanoplatform, we assessed its cytotoxicity against both 4T1 tumor cells and HUVECs using the CCK-8 assay. As shown in Fig. 3A, mPEG-Bi_2_O_3_ exhibited a clear differential cytotoxicity between the 2 cell lines. While the viability of HUVECs remained high across all tested concentrations, 4T1 cells showed a significant, concentration-dependent decrease in viability. Statistical analysis revealed that at concentrations of 50, 100, and 200 μg/ml, the viability of 4T1 cells was significantly lower than that of HUVECs (*P* < 0.05). At the highest concentration (200 μg/ml), 4T1 viability dropped to 63.26 ± 8.62%, whereas HUVEC viability was largely unaffected (94.11 ± 4.37%). These results provide direct and quantitative in vitro evidence that mPEG-Bi_2_O_3_ possesses selective cytotoxicity, effectively inhibiting cancer cells while sparing normal endothelial cells, indicating a promising therapeutic window for its in vivo application.

The interaction of mPEG-Bi_2_O_3_ with 4T1 cells was then assessed. CLSM revealed a time-dependent cellular association of Dil-labeled nanoparticles, with fluorescence localized in the cytoplasm and distinctly separated from DAPI-stained nuclei, suggesting successful internalization (Fig. 3E). This was quantitatively confirmed by flow cytometry, which showed a progressive increase in cellular fluorescence intensity, peaking after 4 h of incubation (Fig. 3F). The effective intracellular delivery was further corroborated by the potent downstream biological effects. Upon LIFU activation, mPEG-Bi_2_O_3_ exerted significant sonodynamic activity. Flow cytometry and DCFH-DA staining demonstrated that the mPEG-Bi_2_O_3_ + LIFU group generated the highest levels of intracellular ROS among all groups (Fig. 3C and D). Concurrently, this treatment led to the most pronounced depletion of intracellular GSH (Fig. 3B and I) and induced the most severe disruption of mitochondrial membrane potential, as evidenced by a shift from red to green fluorescence in JC-1 staining (Fig. 3H). These cascading events—ROS burst, antioxidant depletion, and mitochondrial damage—culminated in substantial cancer cell death. Flow cytometry analysis of apoptosis confirmed that the mPEG-Bi_2_O_3_ + LIFU group induced the highest percentage of apoptotic cells (Fig. 3G). Collectively, these results demonstrate that mPEG-Bi_2_O_3_ is efficiently internalized and acts as a potent sonosensitizer, triggering oxidative stress-mediated apoptosis in cancer cells upon LIFU irradiation.

### In vivo biodistribution, clearance kinetics, and preliminary safety of mPEG-Bi_2_O_3_

**Fig. 4.**
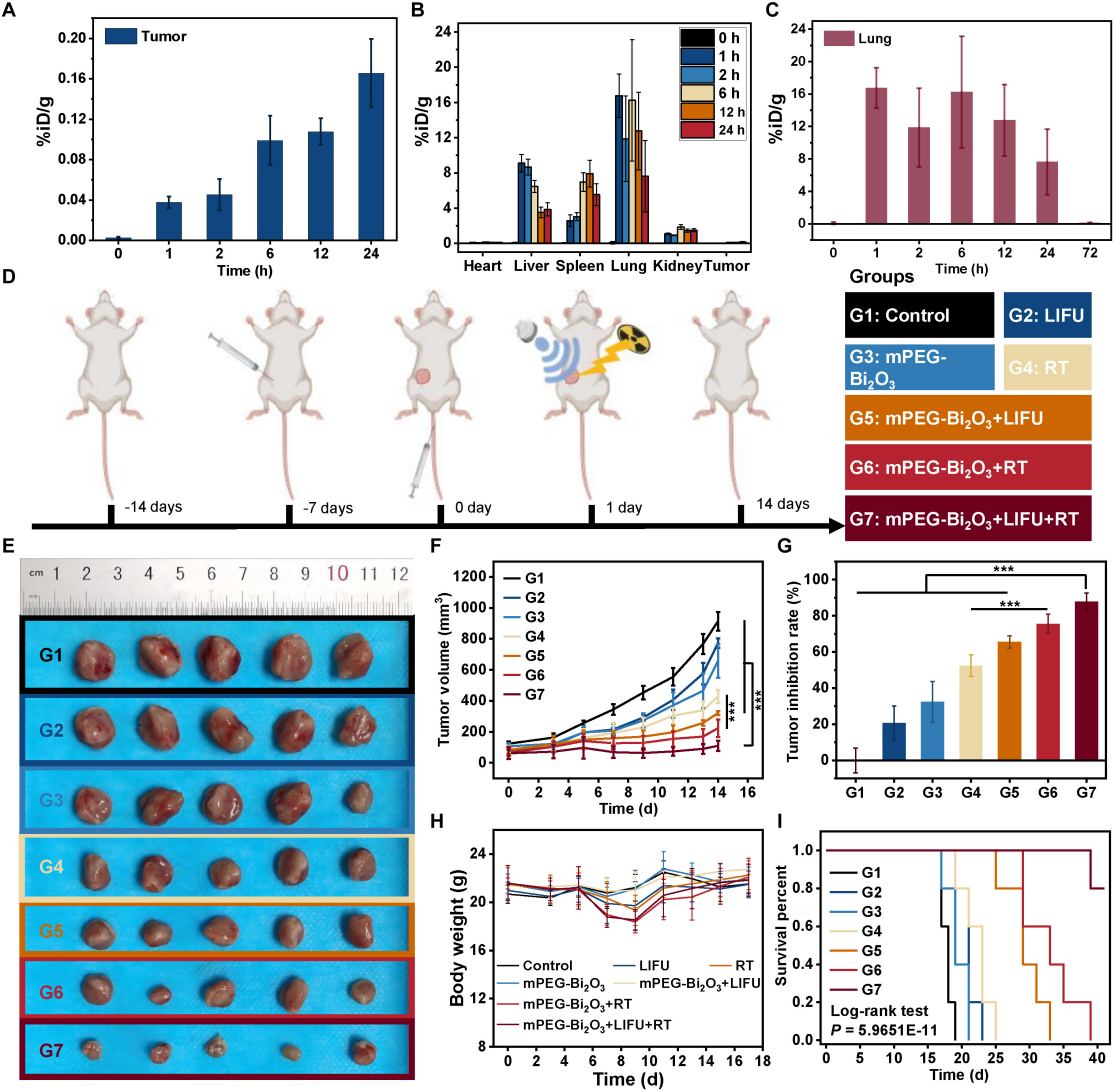
In vivo biodistribution, antitumor efficacy, and pharmacokinetics of mPEG-Bi_2_O_3_. (A) Time-dependent tumor accumulation of mPEG-Bi_2_O_3_ expressed as percentage of injected dose per gram of tissue (%ID/g). (B) Biodistribution of mPEG-Bi_2_O_3_ in major organs from 1 to 24 h post-injection (%ID/g). (C) Clearance kinetics of mPEG-Bi_2_O_3_ in lung tissue over time (%ID/g). (D) Schematic diagram of the mouse treatment protocol. (E) Gross morphology of dissected tumors from different treatment groups. (F) Statistical analysis of tumor volume over time in different groups. (G) Comparison of tumor inhibition rates among different groups. (H) Changes in body weight of tumor-bearing mice across different groups. (I) Kaplan–Meier survival curves. Data are mean ± SD [*n* = 5 biologically independent mice per group for (E) to (I); *n* = 3 mice per time point for (A) to (C)]. *P < 0.05, **P < 0.01, ***P < 0.001; n.s., not significant.

Based on quantitative pharmacokinetic analysis, mPEG-Bi_2_O_3_ exhibited a time-dependent accumulation profile in tumor tissue, peaking at 0.17 ± 0.03 %ID/g at 24 h post-injection (Fig. 4A). In normal tissues, a rapid distribution was observed within 1 h (Fig. 4B), with the highest uptake in the lungs (16.76 ± 2.47 %ID/g), followed by the liver (9.11 ± 1.00 %ID/g), spleen (2.58 ± 0.67 %ID/g), and kidneys (1.08 ± 0.08 %ID/g); the heart showed minimal uptake (0.09 ± 0.17 %ID/g). Clearance kinetic analysis (Fig. 4C) demonstrated that the initially high Bi content in the lungs decreased rapidly, falling below the detection limit (<0.001 %ID/g) within 3 d, corresponding to a reduction of >99.9% from the 1-h level. A concurrent decreasing trend was observed in the liver, spleen, and other organs over the same period.

Quantitative analysis of hematological and serum biochemical parameters at days 1, 14, and 28 revealed that mPEG-Bi_2_O_3_ exhibited a favorable safety profile overall. While most indicators remained within normal physiological ranges without significant intergroup differences, 2 transient variations were noted. First, a dose-dependent elevation in serum lactate dehydrogenase was observed on day 1 post-injection (Fig. S1), likely reflecting acute physiological stress during initial nanoparticle clearance. Second, granulocyte counts showed a modest but statistically significant increase in the high-dose group (40 mg/kg) compared to controls on day 14 (Fig. S2), suggesting a mild, delayed immune stimulation. Critically, both parameters normalized completely by the subsequent time points (days 14/28 for lactate dehydrogenase; day 28 for granulocyte counts), with no sustained differences observed. All other hematological and biochemical markers showed no significant alterations at any time point. H&E staining revealed no evident damage or pathological changes in the heart, liver, spleen, lungs, or kidneys across all groups (Fig. S3). Collectively, these data indicate that mPEG-Bi_2_O_3_ administration may trigger transient, self-limiting physiological responses but does not cause persistent organ toxicity or hematological dysfunction within the 28-d observation period, supporting its favorable preliminary safety profile alongside its efficient tumor targeting and clearance.

### In vivo antitumor effects of mPEG-Bi_2_O_3_ in tumor-bearing mice

Following the intravenous injection of mPEG-Bi_2_O_3_, tumor regions in mice were treated with LIFU and x-ray irradiation according to the schematic protocol (Fig. 4D). Tumor growth dynamics over the 14-day treatment period are shown in Fig. S4, and the gross morphology of excised tumors on day 14 is presented in Fig. 4E. Quantitative analysis of tumor volumes on day 14 revealed significant differences among groups (Fig. 4F). The tumor volume in the control group was 913.20 ± 59.55 mm^3^. In contrast, the combination therapy group (mPEG-Bi_2_O_3_ + LIFU + RT) exhibited the most potent inhibition, with tumor volume reduced to 109.60 ± 33.17 mm^3^, corresponding to a tumor inhibition rate of 87.82 ± 4.77% (*P* < 0.001) (Fig. 4G). This efficacy was significantly superior to that of other treatment groups. Specifically, tumor volumes in the mPEG-Bi_2_O_3_ + RT and mPEG-Bi_2_O_3_ + LIFU groups were 223.40 ± 56.22 mm^3^ (inhibition rate: 75.52 ± 5.29%) and 427.80 ± 42.56 mm^3^ (inhibition rate: 52.42 ± 6.03%), respectively (*P* < 0.001 for all comparisons versus combination therapy) (Fig. 4F and G). Critically, to isolate the radiosensitizing effect of the nanomaterial, we compared the mPEG-Bi_2_O_3_ + RT group with the RT alone group. The tumor inhibition in the mPEG-Bi_2_O_3_ + RT group (75.52 ± 5.29%) was significantly greater than that in the RT alone group (65.50 ± 3.39%) (*P* < 0.05) (Fig. 4G), indicating an independent radiosensitizing effect of the mPEG-Bi_2_O_3_ nanomaterial. Throughout the treatment period, all groups maintained comparable body weights, indicating no significant treatment-related systemic toxicity (Fig. 4H).

To assess the long-term therapeutic benefit, tumor-bearing mice were monitored for survival. Kaplan–Meier survival curves were generated and statistically compared using the log-rank test (Fig. 4I). The median survival of the combination therapy group (mPEG-Bi_2_O_3_ + LIFU + RT) was significantly extended to 41 d, compared to 19, 23, and 33 d for the control, SDT alone, and RT alone groups, respectively. The log-rank test confirmed statistically significant differences between the combination group and all other groups (*P* < 0.001). At the 42-d observational endpoint, the survival rate in the combination group was 80%, whereas all mice in the other groups had died within 39 d. Therefore, mPEG-Bi_2_O_3_ combined with RT and LIFU could significantly enhance the antitumor efficacy and prolong the survival of tumor-bearing mice.

### Antitumor immune response following mPEG-Bi_2_O_3_ treatment

**Fig. 5.**
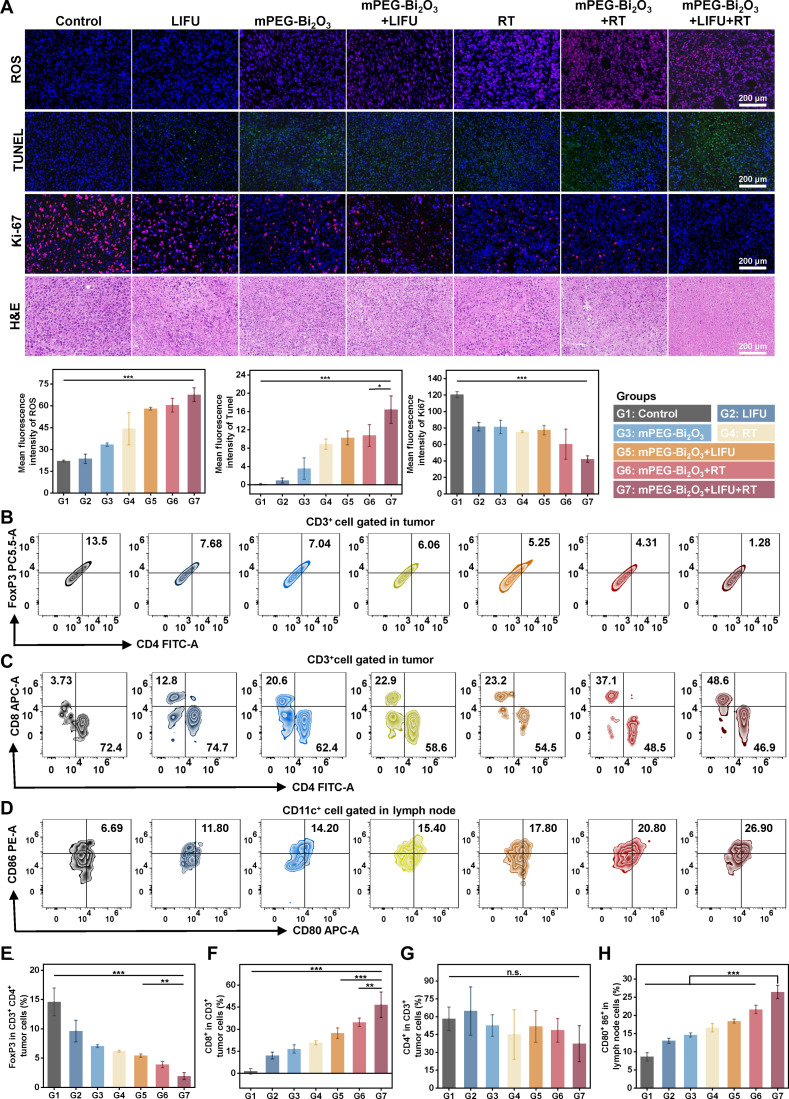
Remodeling of the tumor immune microenvironment by mPEG-Bi_2_O_3_-based combination therapy. (A) Histopathological analysis of tumor tissues 24 h post-treatment. Representative images show ROS detection (DCFH-DA staining, red), apoptosis (TUNEL staining, green), proliferation (Ki-67 staining, red), and tissue morphology (H&E staining). Scale bars, 200 μm. Quantitative analysis of their fluorescence intensity is presented as mean ± SD (*n* = 3 mice). (B) Representative flow cytometry plots and (E) quantitative analysis of regulatory T cells (Tregs, CD3^+^CD4^+^FoxP3^+^) within tumor-infiltrating lymphocytes. (C) Representative flow cytometry plots and quantitative analysis of (F) cytotoxic CD8^+^ T cells and (G) helper CD4^+^ T cells (CD3^+^CD4^+^) within tumor-infiltrating lymphocytes. (D) Representative flow cytometry plots and (H) quantitative analysis of mature DCs (CD11c^+^CD80^+^CD86^+^) in tumor-draining lymph nodes. For all flow cytometry analyses (B to H), data are presented as mean ± SD (*n* = 5 biologically independent mice per group). Statistical significance was determined by one-way ANOVA with Tukey’s post hoc test. *P < 0.05, **P < 0.01, ***P < 0.001; ns, not significant.

Pathological analysis of tumor tissues collected 24 h post-treatment revealed the most pronounced therapeutic effects in the mPEG-Bi_2_O_3_ + LIFU + RT group. This group exhibited the highest level of ROS accumulation (indicated by intense red fluorescence) (*P* < 0.05), extensive necrosis in H&E staining, the largest area of apoptosis in TUNEL staining (*P* < 0.05), and the lowest Ki-67 proliferation index (*P* < 0.05), collectively indicating effective tumor cell death and growth inhibition (Fig. 5A).

To objectively evaluate the immune response elicited by the combination therapy, lymph nodes and tumor tissues were collected on day 14 post-treatment for quantitative flow cytometry analysis of immune cell subsets. The detailed gating strategies are provided in Fig. S5A to C. The results revealed significant remodeling of the tumor immune microenvironment (Fig. 5B to H). The proportion of Tregs (identified as CD3^+^CD4^+^FoxP3^+^) within tumor-infiltrating lymphocytes was significantly reduced, from 14.60 ± 2.35% in the control group to 1.91 ± 0.60% in the combination treatment group (*P* < 0.001) (Fig. 5B and E). Concurrently, the infiltration of cytotoxic T lymphocytes was markedly enhanced, with the percentage of CD8^+^ T cells rising from 1.33 ± 1.85% to 46.60 ± 8.69% (*P* < 0.001) (Fig. 5C and F). The proportion of helper T cells (CD3^+^CD4^+^) showed a decreasing trend but did not reach statistical significance (Fig. 5C and G). At the systemic level, the combination treatment significantly promoted DC maturation in draining lymph nodes. The proportion of mature DCs (CD80^+^CD86^+^) increased from 8.60 ± 1.14% in the control group to 26.40 ± 1.82% (*P* < 0.001) (Fig. 5D and H).

Consistent with the cellular immune profile, cytokine analysis in the tumor microenvironment showed a significant increase in pro-inflammatory factors (TNF-α, IFN-γ, IL-6, and IL-12p70) and a decrease in the anti-inflammatory cytokine IL-10 in the mPEG-Bi_2_O_3_ + LIFU + RT group (Fig. S6). These findings highlight the potential of mPEG-Bi_2_O_3_ in enhancing antitumor immunity by promoting DC maturation and increasing CD8^+^ T proportions while reducing Tregs in the tumor microenvironment.

## Discussion

This study successfully developed an mPEG-Bi_2_O_3_ nanoplatform through a surface engineering strategy. In contrast to recent research trends that focus on enhancing the intrinsic properties of Bi-based materials via complex heterojunctions or elemental doping [1,3,4,9], our work presents a distinct paradigm. We demonstrate that precise surface functionalization with mPEG can effectively address the critical delivery bottlenecks of single-component Bi_2_O_3_, thereby unlocking its full therapeutic potential for synergistic SDT-RT. This approach prioritizes pharmacokinetic performance and in vivo activation, offering a complementary and potentially more translatable path to complex material design.

The successful construction and characterization of mPEG-Bi_2_O_3_ laid the foundation for its high performance. The material exhibited a thin-layered nanosheet structure with a high specific surface area, which provides abundant reactive sites and facilitates efficient charge carrier migration and separation, thereby promoting ROS generation under ultrasound [22–24]. More importantly, detailed XPS analysis confirmed the creation of a defect-rich electronic structure, characterized by a substantial population of oxygen vacancies coupled with mixed Bi^3+^/Bi^5+^ valence states. This configuration is widely recognized to enhance charge separation and serve as active sites for catalytic reactions, which underpins the platform’s exceptional sonocatalytic activity [7,8]. The cornerstone of this design, however, lies in the precise surface engineering. The successful and quantitative grafting of mPEG onto Bi_2_O_3_ was unequivocally verified, a modification that proved pivotal. By imparting a high-density hydrophilic corona, the mPEG coating conferred exceptional colloidal stability upon the nanoplatform in complex physiological media, including serum. This critical attribute—demonstrated by negligible aggregation over extended periods—directly addresses a major translational hurdle. It ensures prolonged blood circulation, minimizes premature clearance, and is fundamentally responsible for the efficient passive tumor targeting and accumulation observed in vivo, which are prerequisites for any effective nanomedicine [5,6].

The core therapeutic mechanism of mPEG-Bi_2_O_3_ is a cascaded process of “oxygen enhancement–ROS burst–antioxidant barrier disruption”, which is substantiated by a series of quantitative experiments. First, kinetic dissolved oxygen monitoring confirmed the platform’s ability to catalytically generate oxygen from endogenous H_2_O_2_ under simulated tumor acidic conditions, effectively alleviating tumor hypoxia (“oxygen enhancement”) [11,12]. Second, ESR spectroscopy with specific spin traps distinctly captured the generation of ·O_2_^−^ and ·OH radicals under LIFU, with the highest signal intensity in the mPEG-Bi_2_O_3_ + LIFU group, providing direct evidence for the “ROS burst” [13,14]. This was further corroborated by TMB oxidation and DPBF degradation assays. Third, the DTNB assay quantitatively demonstrated the time-dependent depletion of GSH, validating the “antioxidant barrier disruption” [15,16]. This interconnected cascade, moving beyond mere description to data-driven inference, overcomes the dual limitations of hypoxia and high antioxidant capacity in the tumor microenvironment.

In vitro studies validated the platform’s functionality and selectivity. mPEG-Bi_2_O_3_ showed significantly lower cytotoxicity toward normal endothelial cells (HUVECs) compared to 4T1 tumor cells, indicating a promising therapeutic window [17,18]. Cellular uptake was confirmed by CLSM and flow cytometry, leading to potent sonodynamic effects. Under LIFU, mPEG-Bi_2_O_3_ induced significant intracellular ROS generation, GSH depletion, mitochondrial membrane potential disruption, and, ultimately, apoptosis [21,23]. This established its role as an efficient sonosensitizer, forming the basis for combination with RT.

In vivo, the combination therapy demonstrated superior efficacy. Quantitative biodistribution data (%ID/g) showed passive tumor targeting with peak accumulation at 24 h, while rapid clearance from major organs (e.g., >99.9% from lungs within 3 d) indicated favorable pharmacokinetics [22,24]. The antitumor effect of the triple therapy (mPEG-Bi_2_O_3_ + LIFU + RT) was significantly more potent than any dual-modality treatment, with an inhibition rate of 87.82 ± 4.77% and a significant extension of survival. Crucially, the mPEG-Bi_2_O_3_ + RT group showed significantly better tumor inhibition than RT alone, directly confirming the nanomaterial’s radiosensitizing effect in vivo. This synergy is attributed to the integration of multiple mechanisms: (a) Bi’s high atomic number enhances local radiation energy deposition [25–30]; (b) LIFU-triggered catalytic oxygen production alleviates hypoxia, a key factor in RT resistance [31–35]; and (c) the combined oxidative stress from SDT and RT creates an amplified cytotoxic effect [36–40].

Furthermore, the treatment effectively remodeled the tumor immune microenvironment. Flow cytometry analysis revealed that the combination therapy significantly promoted DC maturation in lymph nodes, increased tumor-infiltrating cytotoxic CD8^+^ T cells, and suppressed Tregs [27,41]. This was accompanied by a shift in serum cytokines toward a pro-inflammatory profile. These changes indicate that the therapy may activate the DC–T cell immune axis and reverse immunosuppression [42–45], adding a valuable immunomodulatory dimension to the direct tumor-killing effect.

The biosafety profile of mPEG-Bi_2_O_3_ is supported by comprehensive and longitudinal assessments. The observed rapid systemic clearance (e.g., >99.9% from lungs within 3 d) fundamentally limits tissue residence time and is a key pharmacokinetic determinant of its safety. Quantitative analysis over a 28-d period revealed a favorable overall profile: The vast majority of hematological and serum biochemical parameters remained within normal physiological ranges, and no histopathological damage was observed in major organs at any time point, even at a high dose (40 mg/kg), consistent with previous studies [34,35]. Although transient, self-limiting variations in specific markers (lactate dehydrogenase on day 1; granulocyte count on day 14) were noted, these resolved completely and were not associated with any tissue injury. The robust colloidal stability conferred by mPEG is central to this safety outcome, as it minimizes opsonization and rapid clearance by the mononuclear phagocyte system, thereby reducing the risk of nonspecific organ accumulation [5,6]. Therefore, the integrated evidence—rapid clearance kinetics, the absence of persistent organ dysfunction, and the resolution of minor early-phase responses—collectively indicates a low risk of chronic accumulation and long-term toxicity, supporting the biocompatibility of the nanoplatform.

### Limitations and future perspectives

While this study provides substantial evidence for the proposed mechanism and therapeutic efficacy, we acknowledge certain limitations that point to future research directions. First, while the in vivo data robustly demonstrate radiosensitization, detailed in vitro assays such as clonogenic survival or γ-H2AX foci quantification would provide more direct and quantitative molecular-level evidence of the synergy between SDT and RT [46,47]. A more granular molecular-level dissection, such as employing advanced in situ probes for real-time O_2_/ROS imaging and performing detailed kinetic analysis of the catalytic cycle, would further elucidate the spatiotemporal dynamics of the cascade. Second, from a pharmacokinetic and safety perspective, while rapid clearance was observed, future studies should include extended biodistribution monitoring and direct quantification of excretion pathways through ICP-MS analysis of feces and urine to provide a complete picture of the nanoplatform’s in vivo fate and elimination kinetics [48,49]. From a materials science perspective, quantitative analyses of oxygen vacancy concentration and PEG grafting efficiency would provide deeper structure–property insights. These focused investigations will be valuable for optimizing the nanoplatform design and personalizing combination therapy regimens.

### Conclusion

**Fig. 6.**
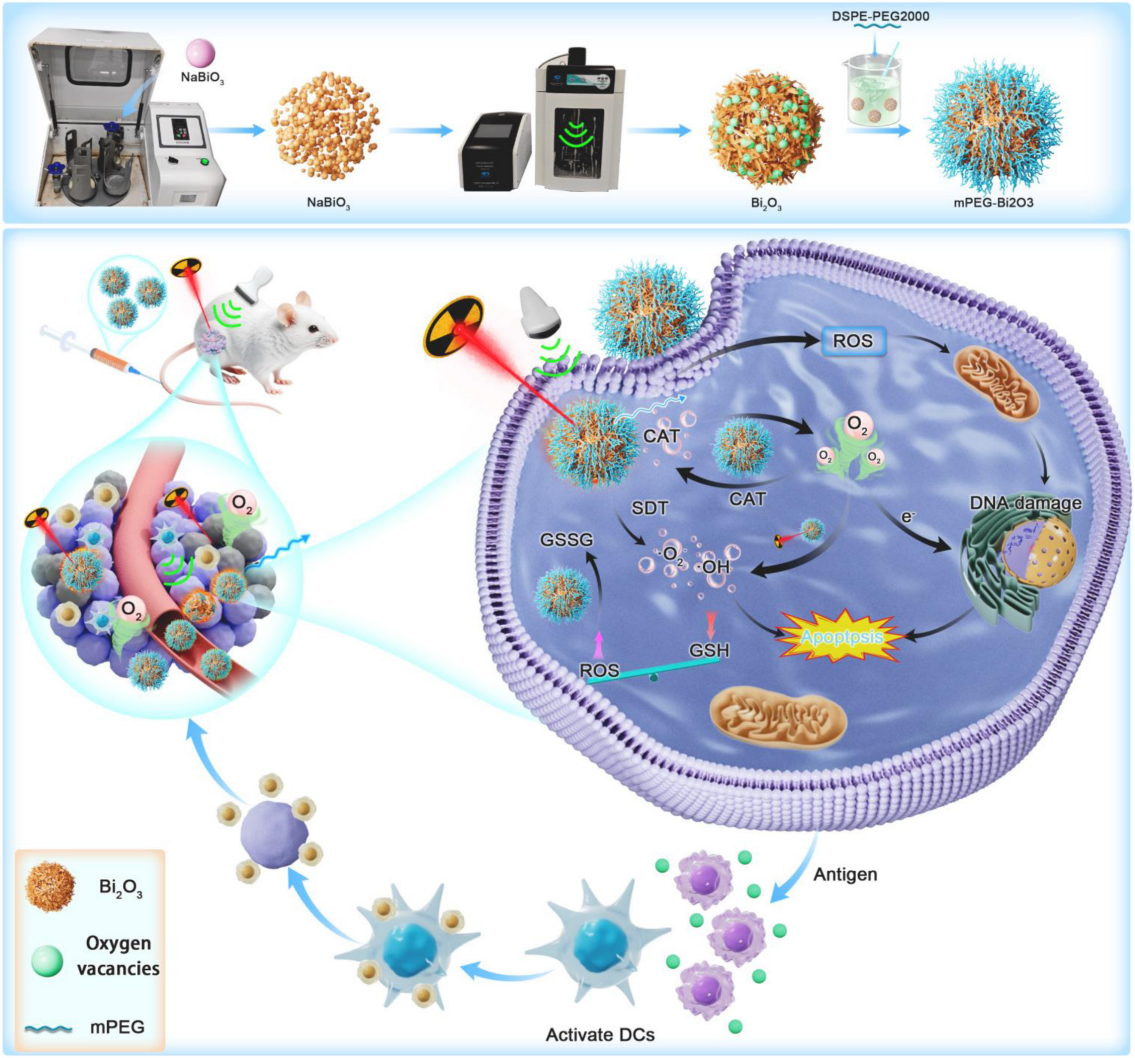
Schematic diagram of the synthesis of mPEG-Bi_2_O_3_ and their in vivo anti-tumor mechanism under LIFU and x-ray irradiation. The synthesis involves vacuum ball milling followed by PEG modification through an ultrasound-assisted liquid-phase exfoliation method, featuring dual functionality of mPEG-Bi_2_O_3_ as both a radiation and sonosensitive agent: (1) Adjusting the bandgap enhances electron-hole separation and boosts ultrasound-induced ROS generation, representing SDT; (2) mPEG-Bi_2_O_3_ achieves RT sensitization due to its high x-ray attenuation coefficient; and (3) the consumption of GSH disrupts the antioxidant barrier, facilitating oxygen supply while exploiting the sensitization properties of Bi to create a tri-dimensional cooperative effect characterized by an “oxygen enhancement–ROS explosion–antioxidant barrier disruption”.

In summary, we developed an mPEG-Bi_2_O_3_ nanoplatform via a surface engineering strategy. This platform synergistically enhances SDT and RT through a cascaded mechanism involving catalytic oxygen production, ROS burst, and GSH depletion (Fig. 6). It demonstrates potent antitumor efficacy coupled with beneficial immune microenvironment remodeling and a favorable safety profile characterized by efficient tumor targeting and rapid systemic clearance. This work demonstrates mPEG-Bi_2_O_3_ as a promising and biocompatible agent for combined SDT-RT cancer treatment.

## Data Availability

Data will be made available on request.
