# Supplementary material for: Enhanced Sonodynamic Therapy and Radiotherapy Efficacy: Modified Polyethylene Glycol–Bismuth Trioxide Nanoplatform for Targeted Tumor Treatment
Source: Biomater Res. 2026 Feb 18;30:0325. doi: 10.34133/bmr.0325 (PMC12914061; doi:10.34133/bmr.0325)
Supplement: Supplementary 1 — Figs. S1 to S6 [file bmr.0325.f1.zip › sm.docx]

SUPPLEMENTARY MATERIALS

Figures S1 to S6

**
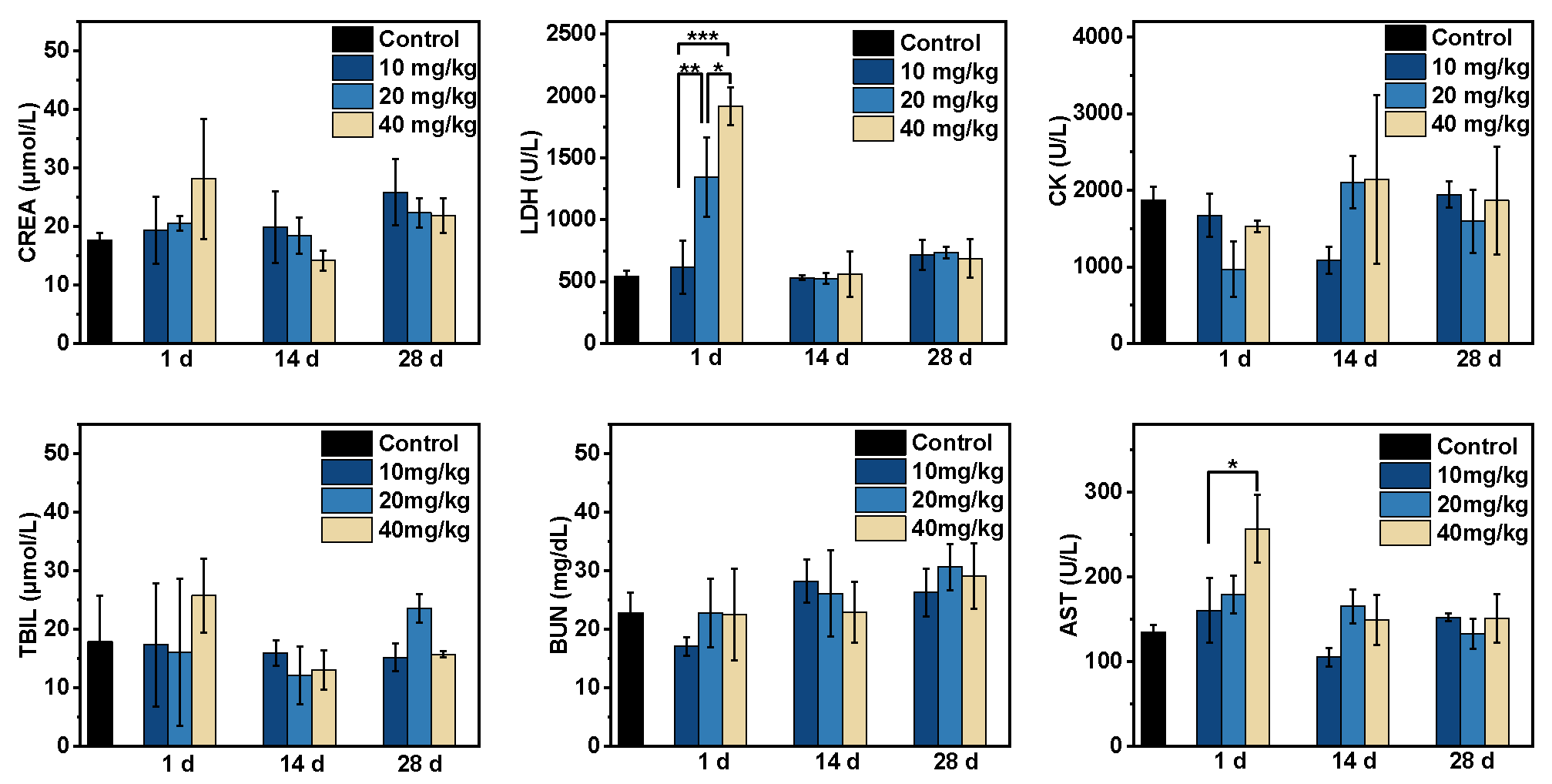
**

**Fig. S1.** Blood biochemical results. Healthy BALB/c mice were tail vein injected with different doses of mPEG-Bi₂O₃ (10, 20, and 40 mg/kg). The control group of mice was given 200 μL of PBS using the same administration method. Blood samples were collected on days 1, 14, and 28 for biochemical testing. CREA, creatinine; LDH, lactate dehydrogenase; CK, creatine kinase; TBIL, total bilirubin; BUN, blood urea nitrogen; AST, aspartate aminotransferase. Data are presented as mean ± SD (n = 3 mice per group per time point). Statistical significance was determined by one-way ANOVA followed by Tukey’s post hoc test. A transient, dose-dependent increase in serum LDH levels was observed on day 1 (*P < 0.05, **P < 0.01, ***P < 0.001). This elevation was self-limiting, as LDH levels returned to baseline and showed no significant differences among groups by days 14 and 28. All other biochemical parameters showed no statistically significant differences compared to the control group at any time point.

**
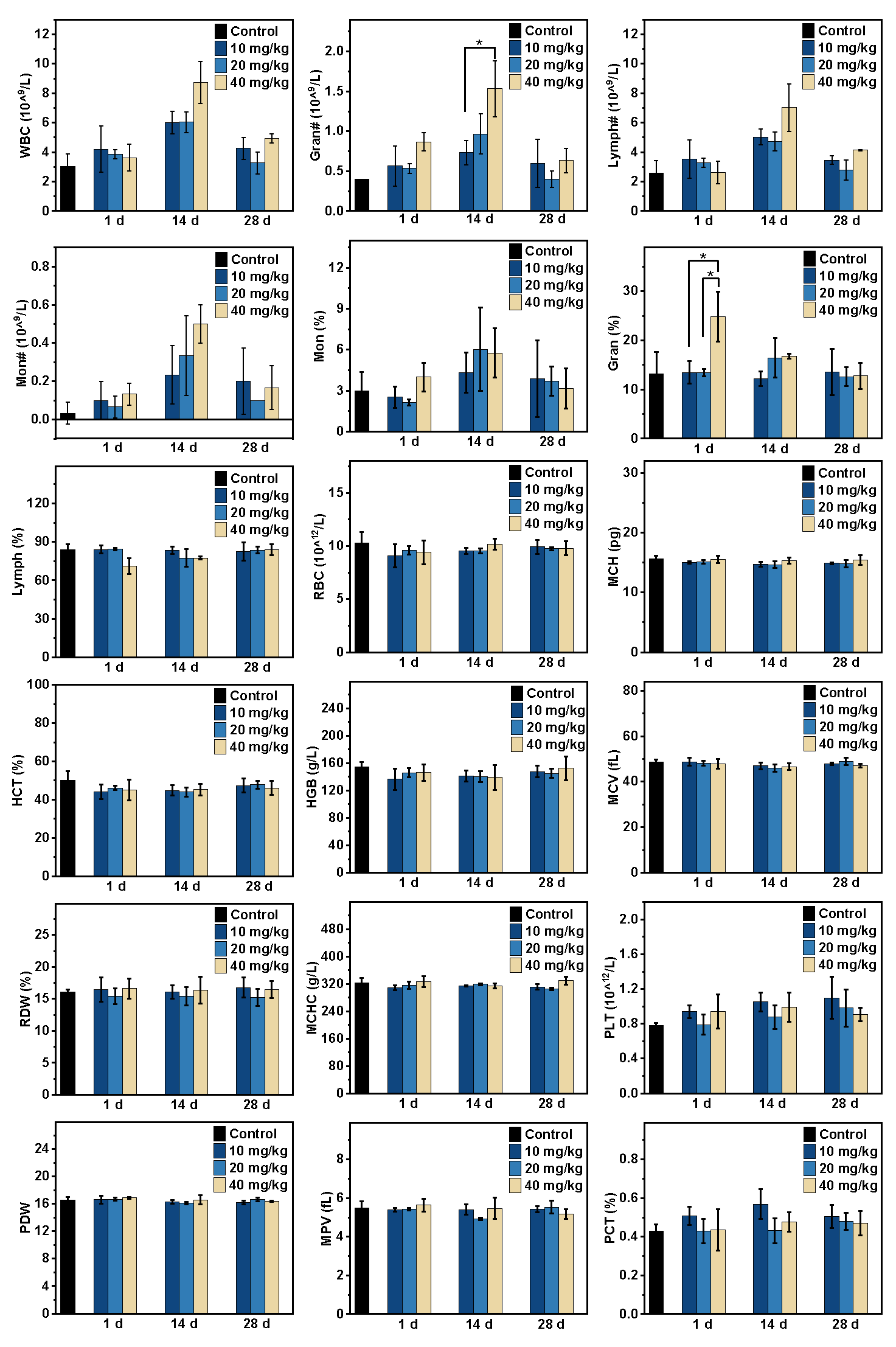
**

**Fig. S2.** Blood routine results. Healthy BALB/c mice were tail vein injected with different doses of mPEG-Bi₂O₃ (10, 20, and 40 mg/kg). The control group of mice was given 200 μL of PBS using the same administration method. Blood samples were collected on days 1, 14, and 28 for hematological analysis. WBC, white blood cell; Gran#, granulocyte absolute count; Lymph#, lymphocyte absolute count; Mon#, monocyte absolute count; Mon%, monocyte percentage; Gran%, granulocyte percentage; Lymph%, lymphocyte percentage; RBC, red blood cell; MCH, mean corpuscular hemoglobin; HCT, hematocrit; HGB, hemoglobin concentration; MCV, mean corpuscular volume; RDW, red cell distribution width; MCHC, mean corpuscular hemoglobin concentration; PLT, platelet count; PDW, platelet distribution width; MPV, mean platelet volume; PCT, plateletcrit. Data are presented as mean ± SD (n = 3 mice per group per time point). Statistical significance was determined by one-way ANOVA followed by Tukey’s post hoc test. A modest but statistically significant increase in Gran# was observed in the high-dose (40 mg/kg) group compared to the control group on day 14 (*P < 0.05). This difference was resolved completely by day 28. All other hematological parameters showed no statistically significant alterations compared to the control group at any time point.

**
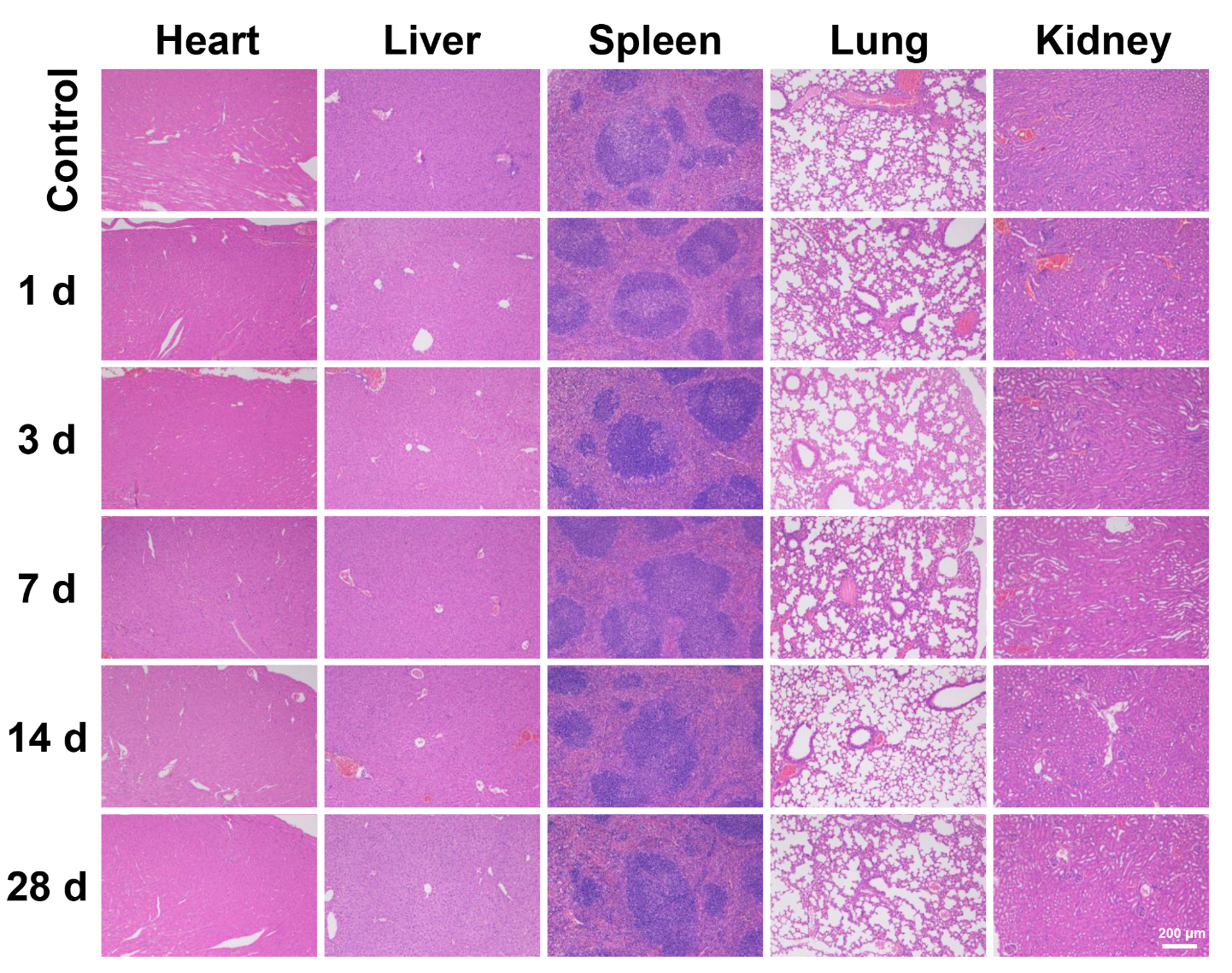
**

**Fig. S3.** H&E staining images of mouse heart, liver, spleen, lungs, and kidneys on days 0, 1, 3, 7, 14, and 28. Scale bar: 200 μm.


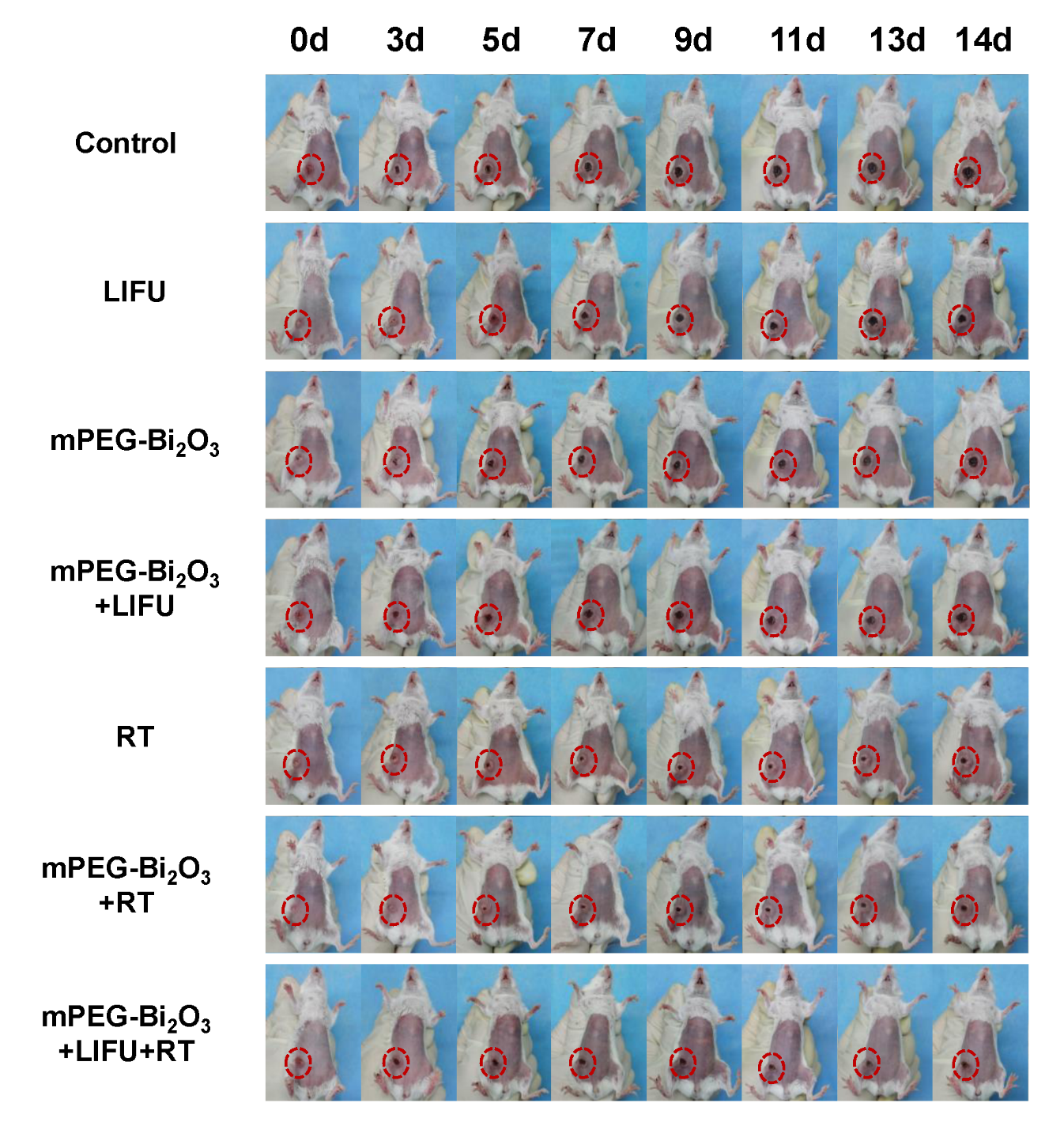


**Fig. S4.** Representative images of 4T1 tumor-bearing mice after treatment in each group. The subcutaneous tumor regions are outlined with red dotted circles.


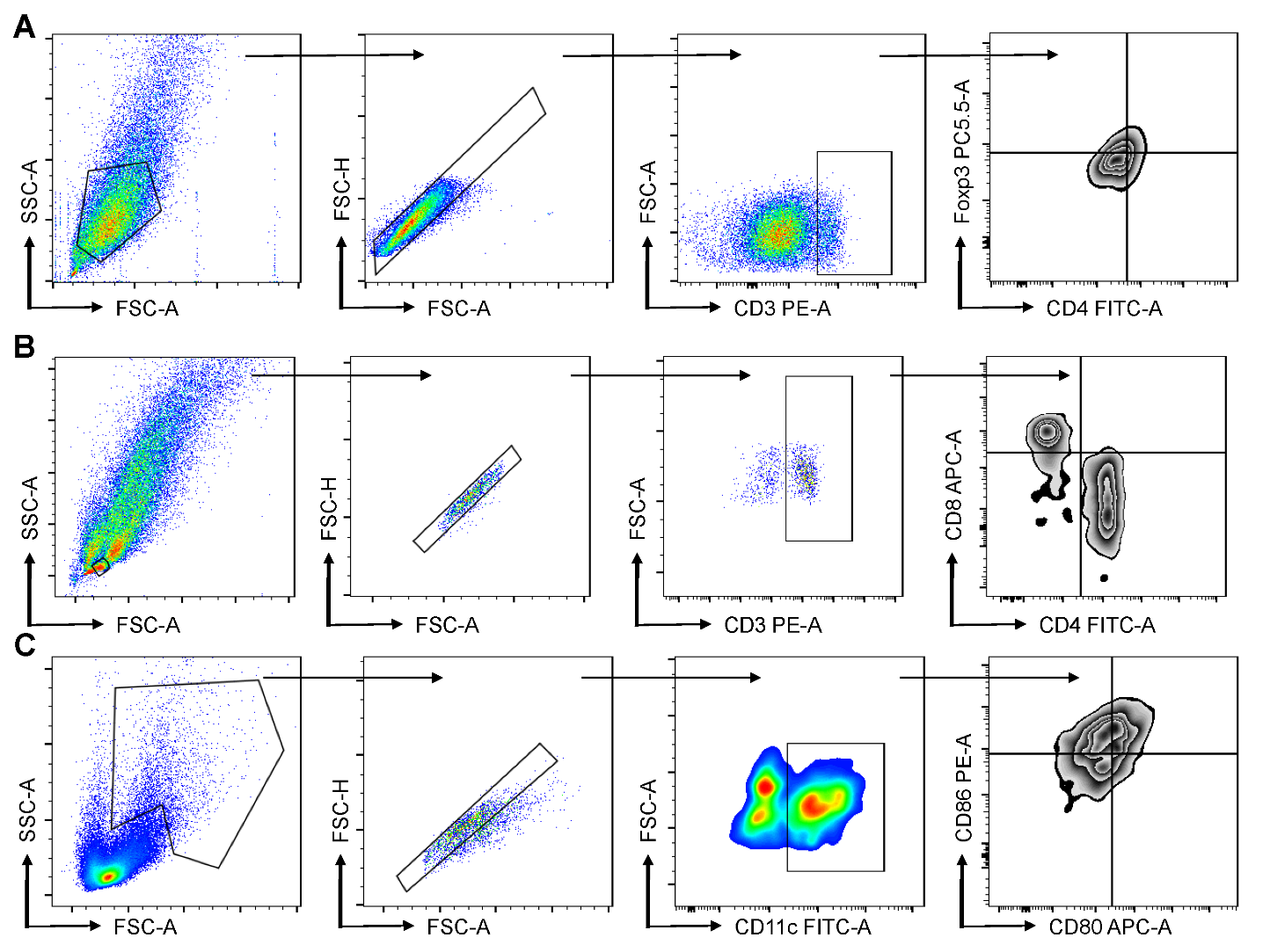


**Fig. S5.** Gating strategies for flow cytometric analysis of immune cell subsets. (A) Gating strategy for Tregs in tumor tissue. Cells were first gated on live, single lymphocytes based on forward scatter (FSC) and side scatter (SSC) properties. From the live single-cell population, T lymphocytes were identified as CD3^+^ cells. Helper T cells (CD4^+^) were then selected from the CD3^+^ gate. Intracellular staining for the transcription factor FoxP3 was performed to identify the Treg subset, defined as CD3^+^CD4^+^FoxP3^+^ cells. (B) Gating strategy for CD8^+^ and CD4^+^ T cells in tumor tissue. Tumor-infiltrating lymphocytes were isolated and gated for live, single cells (as described in A). From this population, T lymphocytes were identified as CD3^+^ cells. Helper T cells (CD4^+^) and cytotoxic T cells (CD8^+^) were then distinguished based on the expression of CD4 and CD8 within the CD3^+^ T cell gate. (C) Gating strategy for mature DCs in lymph nodes. Cells were first gated on live, single lymphocytes based on FSC and SSC properties. From the live single-cell population, DCs were selected as CD11c^+^ cells. Mature DCs were subsequently defined as the population co-expressing the activation markers CD80 and CD86 within the CD11c^+^ gate.

**
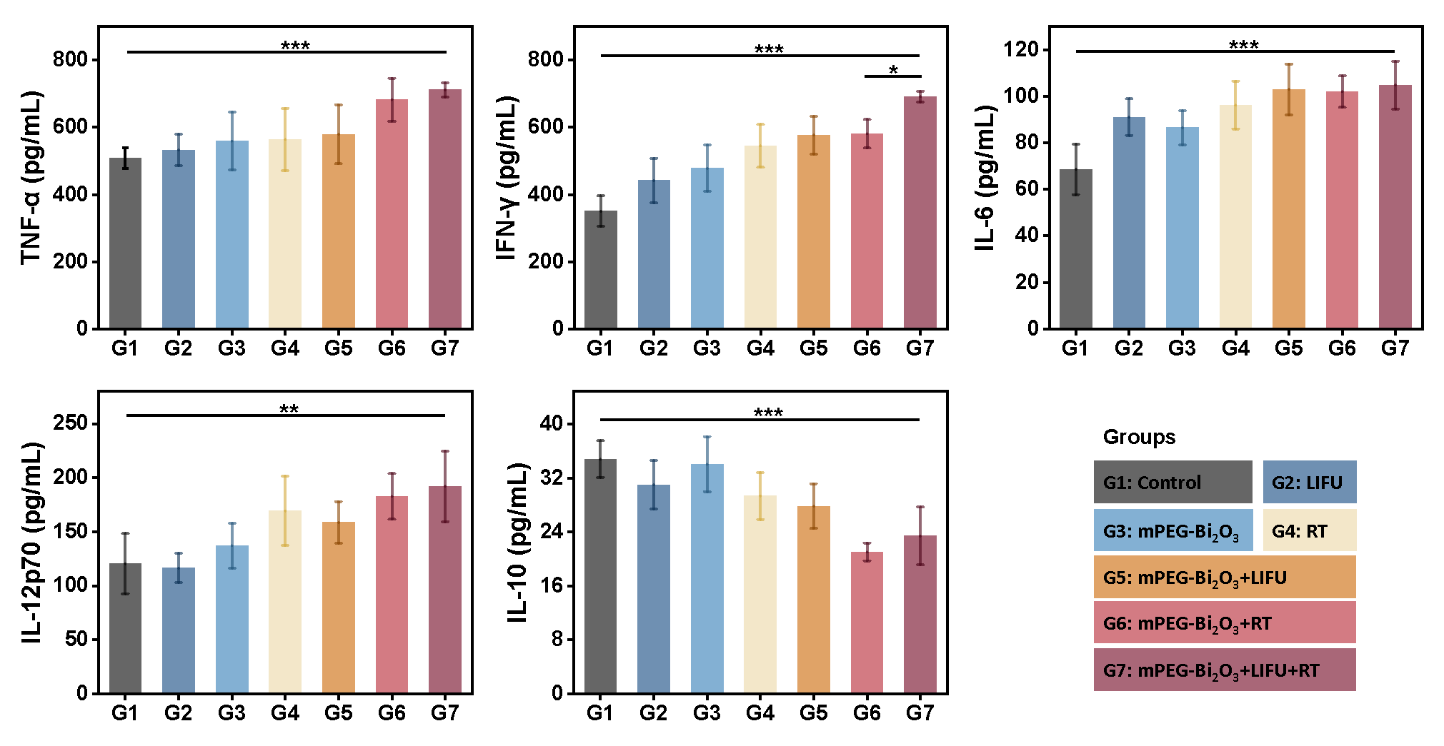
**

**Fig. S6.** ELISA measurement of TNF-α, TNF-γ, IL-6, IL-12p70, and IL-10 expression. Data are presented as mean ± SD (n = 5 mice per group). Statistical significance was determined by one-way ANOVA with Tukey’s post hoc test. **P*<0.05, ***P*<0.01, ****P*<0.001.
