# Supplementary figures and images for: Enhanced Sonodynamic Therapy and Radiotherapy Efficacy: Modified Polyethylene Glycol–Bismuth Trioxide Nanoplatform for Targeted Tumor Treatment
Source: Biomater Res. 2026 Feb 18;30:0325. doi: 10.34133/bmr.0325 (PMC12914061; doi:10.34133/bmr.0325)

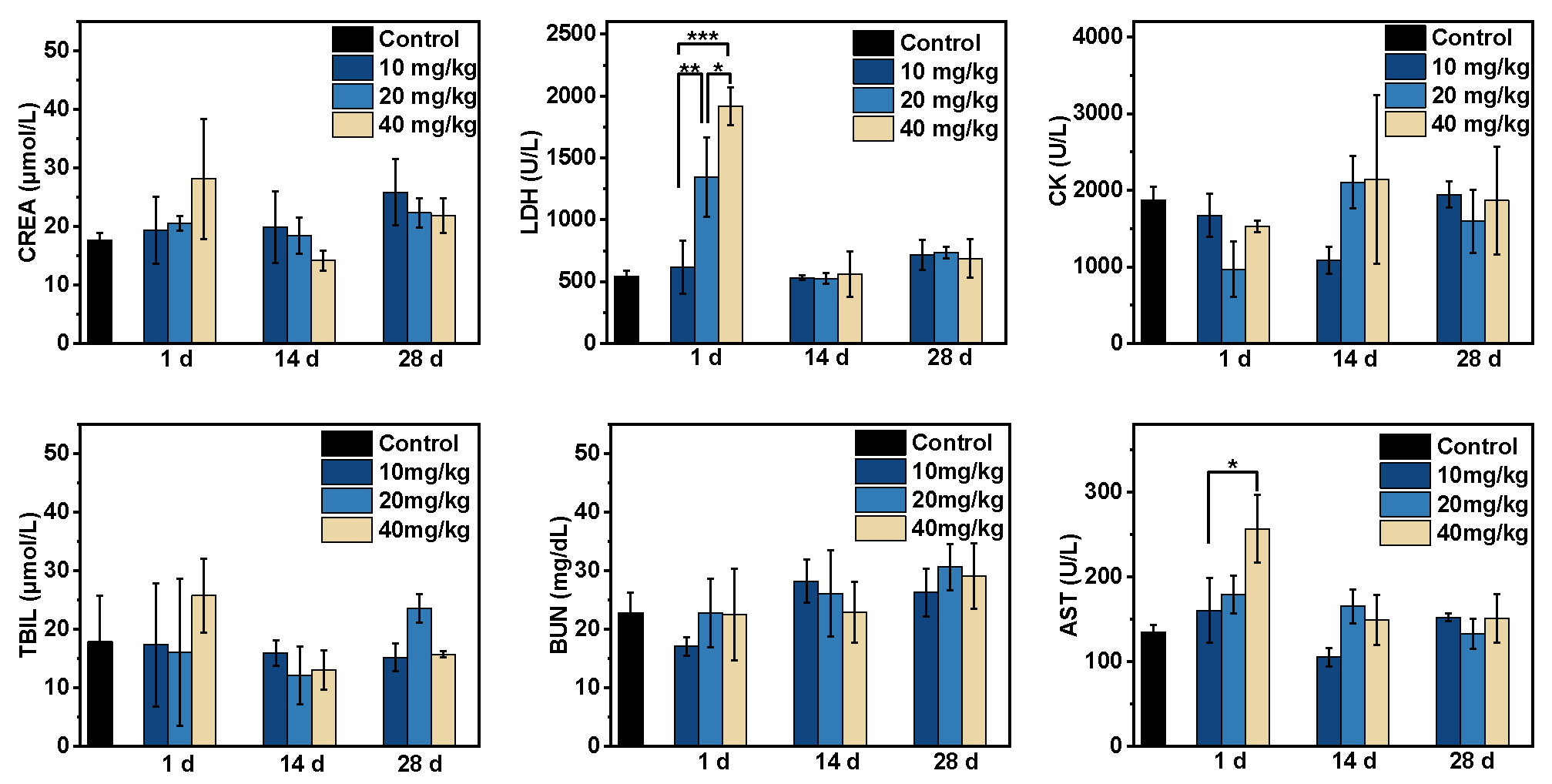

Supplement: Supplementary 1 — Figs. S1 to S6 [file bmr.0325.f1.zip › Supplementary Figure S1 revised.tif]

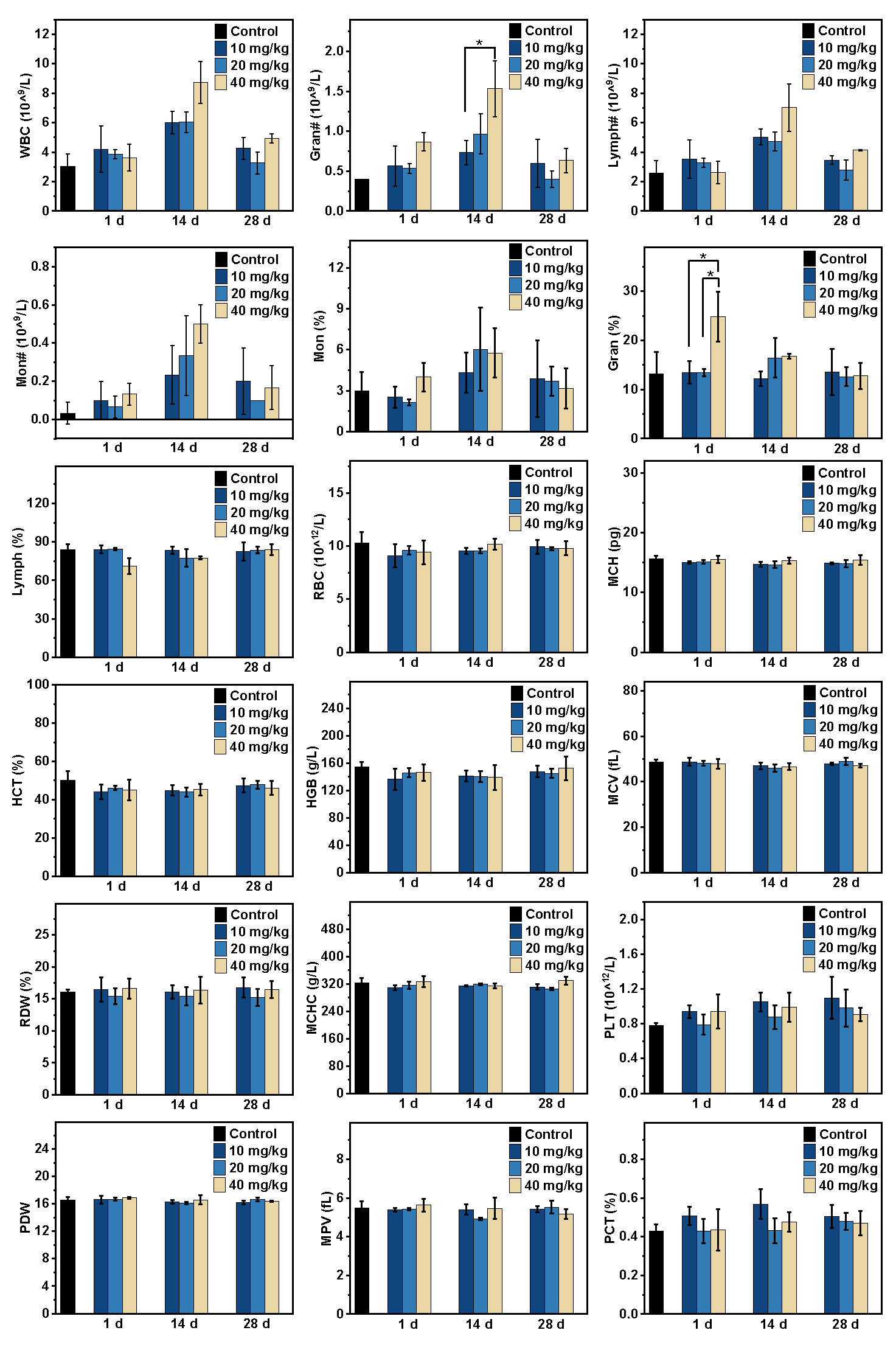

Supplement: Supplementary 1 — Figs. S1 to S6 [file bmr.0325.f1.zip › Supplementary Figure S2 revised.tif]

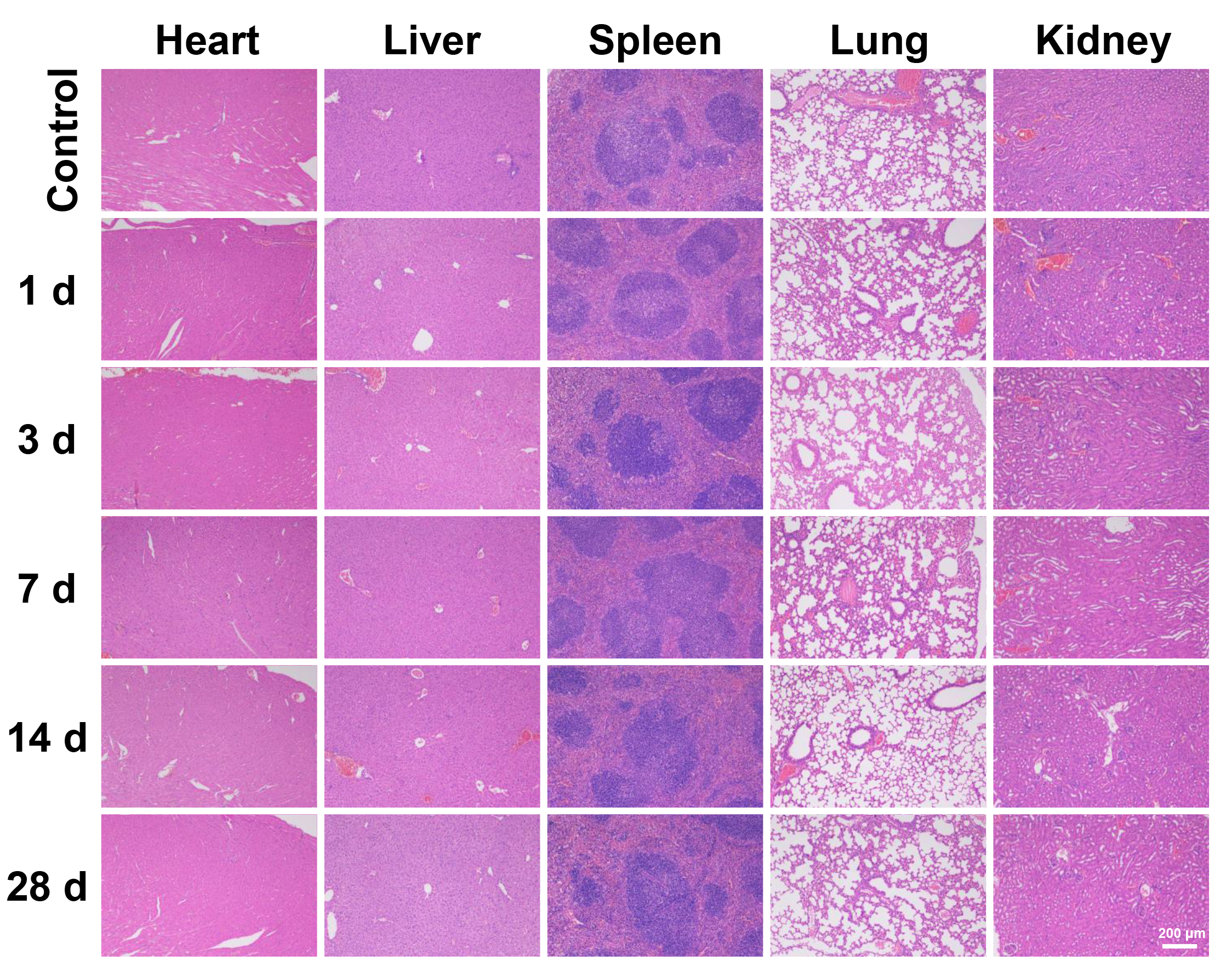

Supplement: Supplementary 1 — Figs. S1 to S6 [file bmr.0325.f1.zip › Supplementary Figure S3.tif]

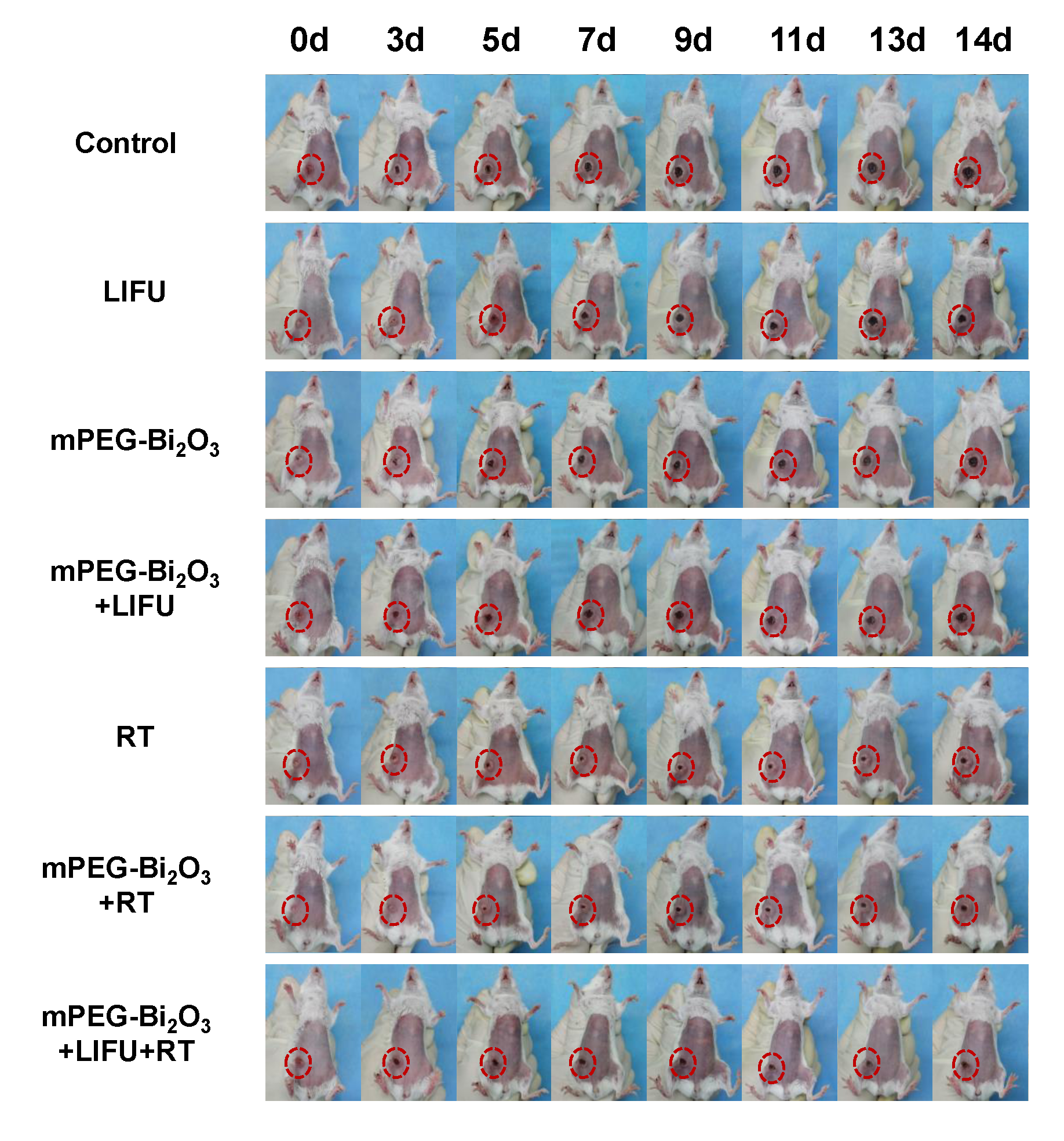

Supplement: Supplementary 1 — Figs. S1 to S6 [file bmr.0325.f1.zip › Supplementary Figure S4 revised.tif]

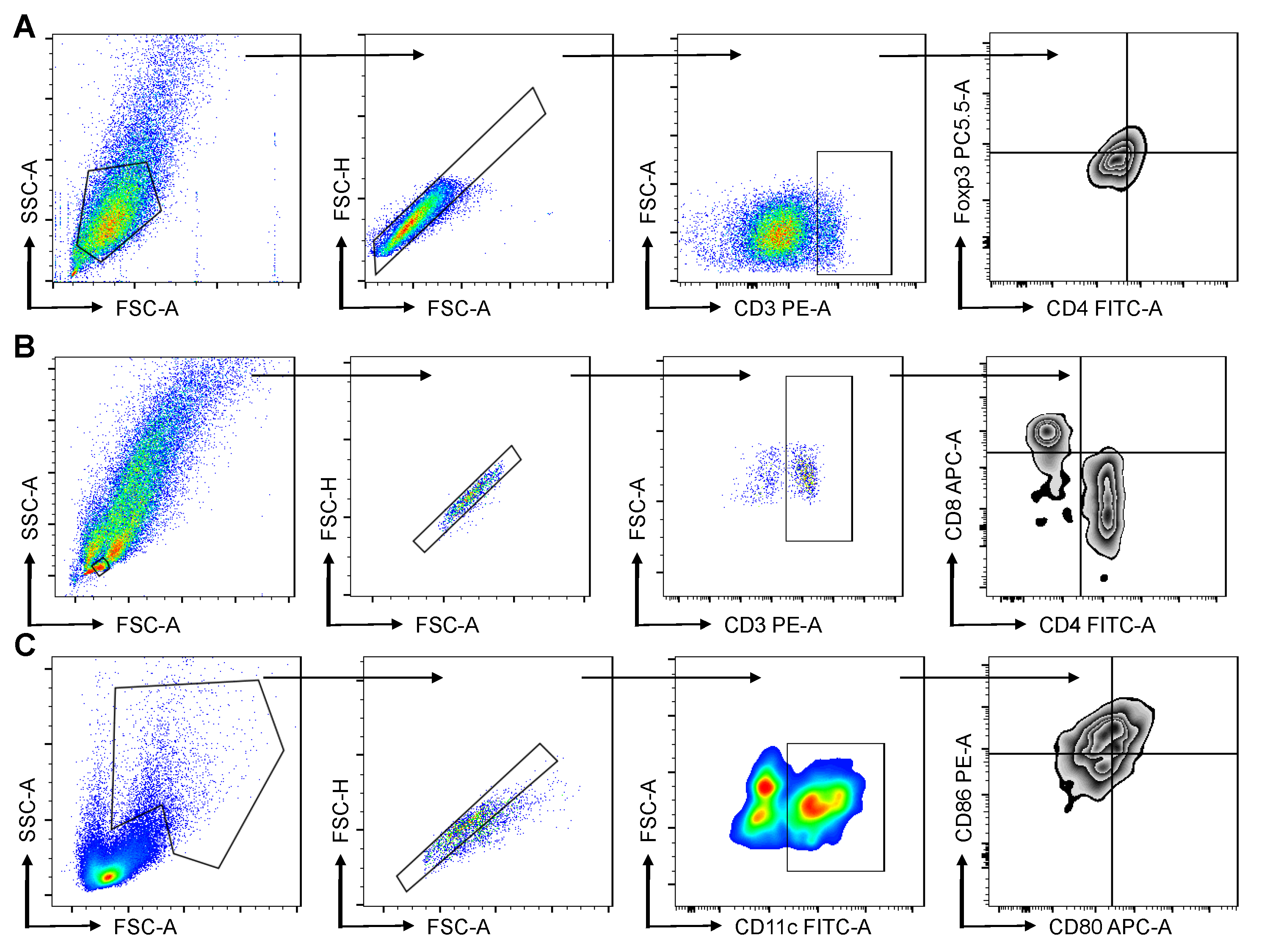

Supplement: Supplementary 1 — Figs. S1 to S6 [file bmr.0325.f1.zip › Supplementary Figure S5 revised.tif]

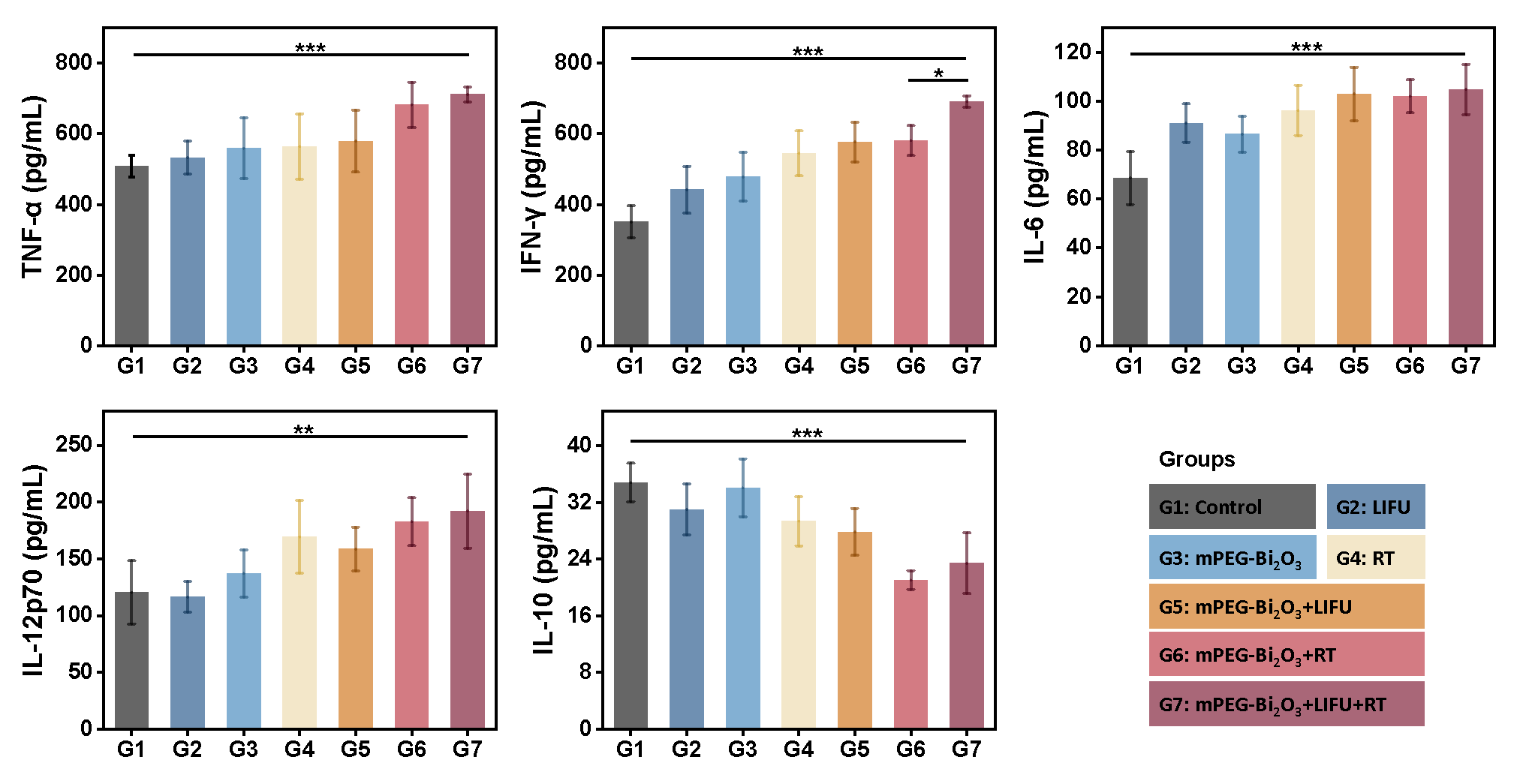

Supplement: Supplementary 1 — Figs. S1 to S6 [file bmr.0325.f1.zip › Supplementary Figure S6 revised.tif]
